# Does Prenatal Stress Shape Postnatal Resilience? – An Epigenome-Wide Study on Violence and Mental Health in Humans

**DOI:** 10.3389/fgene.2019.00269

**Published:** 2019-04-16

**Authors:** Fernanda Serpeloni, Karl M. Radtke, Tobias Hecker, Johanna Sill, Vanja Vukojevic, Simone G. de Assis, Maggie Schauer, Thomas Elbert, Daniel Nätt

**Affiliations:** ^1^Clinical Psychology and Neuropsychology, Department of Psychology, University of Konstanz, Konstanz, Germany; ^2^Department of Studies in Violence and Health Jorge Careli, National School of Public Health of Rio de Janeiro – National Institute of Women, Children and Adolescents Health Fernandes Figueira, Oswaldo Cruz Foundation, Rio de Janeiro, Brazil; ^3^Evolutionary Biology and Zoology, Department of Biology, University of Konstanz, Konstanz, Germany; ^4^Clinical Psychology and Psychotherapy, Department of Psychology, Bielefeld University, Bielefeld, Germany; ^5^Division of Molecular Neuroscience, Department of Psychology, University of Basel, Basel, Switzerland; ^6^Department of Clinical and Experimental Medicine, Center for Social and Affective Neuroscience, Linköping University, Linköping, Sweden

**Keywords:** prenatal stress, intimate partner violence, NR3C1, FKBP5, psychiatric resilience, DNA methylation, retrotransposon, heterochromatin

## Abstract

Stress during pregnancy widely associates with epigenetic changes and psychiatric problems during childhood. Animal studies, however, show that under specific postnatal conditions prenatal stress may have other, less detrimental consequences for the offspring. Here, we studied mental health and epigenome-wide DNA methylation in saliva following intimate partner violence (IPV) during pregnancy in São Gonçalo, a Brazilian city with high levels of violence. Not surprisingly, mothers exposed to pregnancy IPV expressed elevated depression, PTSD and anxiety symptoms. Children had similar psychiatric problems when they experienced maternal IPV after being born. More surprisingly, when maternal IPV occurred both during (prenatal) and after pregnancy these problems were absent. Following prenatal IPV, genomic sites in genes encoding the glucocorticoid receptor (*NR3C1*) and its repressor FKBP51 (*FKBP5*) were among the most differentially methylated and indicated an enhanced ability to terminate hormonal stress responses in prenatally stressed children. These children also showed more DNA methylation in heterochromatin-like regions, which previously has been associated with stress/disease resilience. A similar relationship was seen in prenatally stressed middle-eastern refugees of the same age as the São Gonçalo children but exposed to postnatal war-related violence. While our study is limited in location and sample size, it provides novel insights on how prenatal stress may epigenetically shape resilience in humans, possibly through interactions with the postnatal environment. This translates animal findings and emphasizes the importance to account for population differences when studying how early life gene–environment interactions affects mental health.

## Introduction

Psychological stress during pregnancy, like maternal depression and intimate partner violence (IPV), has widely been associated with detrimental effects in the child (Monk et al., 2012). Effects on child mental health over-represent the literature, such as increased risk of psychiatric problems like attention deficit hyperactivity disorder, autism spectrum disorder, schizophrenia, major depression and anxiety related disorders (O’Connor et al., 2005; Lupien et al., 2009; Fine et al., 2014; Babenko et al., 2015; O’Donnell et al., 2017). However, not all individuals exposed to stress develop stress-related problems. Many appear to have psychiatric resilience, showing no or better outcomes despite severe stress exposures (Rutter, 1999; Russo et al., 2012).

In animals, prenatal stress sometimes leads to general shifts toward what appears to be adaptations in the offspring to the stressful environments (Bateson et al., 2014). In these studies outcome is highly dependent on the postnatal environment, where for example prenatal stress in rodents leads to behavioral changes that are beneficial in predator rich environments but not otherwise (St-Cyr and McGowan, 2015). Analogies have been reported in several taxa including primates, but in humans they remain speculative in nature (Sheriff et al., 2009; Giesing et al., 2010; Nettle et al., 2013; Berghänel et al., 2016). The best human example can be seen in infants consistently exposed to maternal depression, both pre- and postnatally, which are protected against the negative mental effects seen in infants of mothers with depression either pre- or postnatally (but not during both periods) (Sandman et al., 2012). This indicates that an interaction between the pre- and postnatal environments may shape mental health also in humans. A reason why evidence is mostly lacking in humans could therefore be that prenatal stress studies rarely target postnatal exposures in environments with high levels of stress. Growing up in communities characterized by poverty, crime and violence predicts stress related disorders such as post-traumatic stress disorder (PTSD), depression and conduct problems (Elbert and Schauer, 2002; Fowler et al., 2009). Studying the consequences of prenatal stress in communities with high levels of violence could therefore yield unique insights into the interactions between the pre- and postnatal environments, insights that could be particularly important for understanding human mental disorders.

Epigenetic mechanisms, such as DNA methylation, are important for reprogramming the genome following early life stress (Oberlander et al., 2008; Monk et al., 2012; Babenko et al., 2015; Serpeloni et al., 2016; Vangeel et al., 2017), and are believed to shape psychiatric resilience (Rutter et al., 2006). A well-known example is the epigenetic regulation of the glucocorticoid receptor (GR; gene: *NR3C1*) and its chaperone FK506 binding proteins 51 (FKBP51; gene: *FKBP5*). Both proteins have been studied extensively for their involvement in the hypothalamic–pituitary–adrenal (HPA) axis, a physiological pathway that mediates key elements of the stress response. GR uses the stress hormone cortisol as a ligand, and when activated represses the stress response, while FKBP51 exerts a short-loop feedback by inhibiting the nuclear translocation of GR (Zannas and Binder, 2014). Epigenetic dysregulation of *NR3C1* and *FKBP5* have repeatedly been observed in psychiatric disorders following early life stress (Binder et al., 2008; McGowan et al., 2009; Spijker and van Rossum, 2012; Klengel et al., 2013). The reprogramming of specific genes may, however, only represent pieces of the full epigenome-wide consequence of stress (Hunter et al., 2015). Cortisol exposure in young children is, for example, associated with genome-wide loss of DNA methylation in repetitive heterochromatin-like regions, which are genomic regions normally silenced by DNA methylation (Nätt et al., 2015). How stress-related genes and epigenome-wide mechanisms interact in shaping human mental health is poorly understood.

Here, we aimed to investigate the psychiatric and epigenome-wide consequences of prenatal stress, in the form of IPV during pregnancy, in a human population exposed to high levels of violence. IPV affects one third of the women worldwide (Devries et al., 2013). In the present study, we targeted women and children in São Gonçalo, a city located in Rio de Janeiro state in Brazil. São Gonçalo’s population exceeds one million, with high proportion of low-income families and high levels of community/domestic violence (CDV) (Assis et al., 2009). In a previous study targeting the same population, we showed that the epigenome-wide consequences of IPV and CDV in this population can be detected in grandchildren of grandmothers exposed to violence (Serpeloni et al., 2017). Here, we focus on the more immediate epigenetic and psychiatric consequences of IPV, by obtaining epigenome-wide saliva DNA methylation and psychiatric profiles of children exposed to prenatal IPV. We hypothesized that exposure to prenatal IPV in the high violence communities of São Gonçalo will have different consequences than what has been reported in studies of less violent communities.

## Materials and Methods

### Participants

São Gonçalo subjects, 122 grandmothers, 122 mothers, and 120 children were recruited via the “Family strategy program” of the city of São Gonçalo (Brazil) as described before (Serpeloni et al., 2017). Children (46% male) mean age were 13.1 (range = 8–18); mothers 37.9 (range = 25–51); grandmothers 64.0 (range 46–88) years. Sociodemographic variables of mothers and children can be found in Table 1.

**Table 1:** Selected psychiatric variables and social risk factors in the São Gonçalo subsamples.

	Mothers (*n* = 122)		Children (*n* = 120)								
	Pregnancy IPV ^a^		Prenatal IPV ^a^		Trauma ^b^		CDV ^c^		IPV+ (*n* = 21)	IPV- (*n* = 99)	
						
	ρ*	p	ρ*	p	ρ*	*p*	ρ*	p	Mean (SE) or %		*P*-val; χ2 or ANOVA
**Sociodemographic**											
Age (years)	0.003	n.s.	–0.07	n.s.	0.20	<0.05	0.28	<0.01	13.38 (0.54)	13.66 (0.26)	n.s.
Sex (female)	__	__	0.11	n.s.	0.04	n.s.	–0.12	n.s.	0.62	0.52	n.s.
Maternal age birth	–0.11	n.s.	0.01	n.s.	–0.16	n.s.	–0.20	<0.05	24.06 (1.38)	24.97 (0.63)	n.s.
Family income (USD)	–0.11	n.s.	–0.13	n.s.	–0.23	<0.05	–0.13	n.s.	349.87 (51.51)	490.40 (33.26)	n.s.
Family status (married)	–0.07	n.s.	–0.11	n.s.	–0.09	n.s.	–0.13	n.s.	0.61	0.7	n.s.
Education (years)	–0.21	<0.05	–0.10	n.s.	0.03	n.s.	0.15	n.s.	7.20 (0.61)	7.66 (0.26)	n.s.
Maternal care (high) ^d^	0.15	n.s.	0.15	n.s.	–0.01	n.s.	–0.12	n.s.	0.9	0.69	n.s.
Maternal smoking (never) ^§^	–0.09	n.s.	–0.03	n.s.	0.13	n.s.	0.08	n.s.	0.86	0.88	n.s.
Maternal alcohol (never) ^§§^	0.05	n.s.	–0.03	n.s.	0.18	=0.05	0.18	<0.05	0.95	0.91	n.s.
**Stress**											
Trauma ^b^	0.33	<0.001	0.07	n.s.	1	__	0.6	<0.001	2.05 (0.32)	2.15 (0.21)	n.s.
CDV ^c^	0.22	<0.05	0.02	n.s.	0.6	<0.001	1	__	12.90 (2.19)	12.16 (0.90)	n.s.
Prenatal IPV ^a^	0.06	n.s. ^#^	1	__	0.07	n.s.	0.02	n.s.	__	__	__
Pregnancy IPV (maternal) ^a^	1	__	1	__	0.07	n.s.	0.02	n.s.	__	__	__
IPV lifetime (maternal) ^a^	0.55	<0.001	0.56	<0.001	0.28	<0.01	0.26	<0.01	__	__	__
**Psychiatric**											
PTSD severity ^e,f^	0.22	<0.05	0.17	n.s.	0.42	<0.001	0.33	<0.001	6.71 (1.93)	6.13 (0.87)	n.s.
Depression ^g^	0.28	<0.01	–0.04	n.s.	0.34	<0.001	0.32	<0.001	1.55 (0.55)	1.77 0.32)	n.s.
Conduct problems^h^	__	__	–0.06	n.s.	0.18	n.s.	0.21	<0.05	3.25 (0.48)	3.41 (0.19)	n.s.
Hyperactivity ^i^	__	__	0.07	n.s.	0.01	n.s.	0.05	n.s.	4.60 (0.62)	4.35 (0.24)	n.s.
Emotional problems ^j^	__	__	0.22	<0.05	0.24	<0.01	0.25	<0.01	5.38 (0.53)	4.36 (0.27)	n.s.
Peer problems ^k^	__	__	–0.05	n.s.	0.02	n.s.	0.07	n.s.	1.86 (0.32)	2.04 (0.17)	n.s.
Anxiety ^l^	0.19	<0.05	__	__	__	__	__	


*Not all interviewed mothers were mothers to the interviewed children, and vice versa. Of the reported numbers, 115 represent family dyads. Spearman rank correlation. Marital status represents the following categories: Single = 22, Married = 85, Divorced = 13, Widower = 2. ^§^ Categories of pregnancy smoking: Always = 5, Many times = 2, Few times = 4, Never = 111. ^§§^ Categories of pregnancy alcohol use: Always = 3, Many times = 3, Few times = 11, Never = 105. ^#^ Pregnancy IPV exposure in grandmothers did not correlate with the mothers’ experiences of pregnancy IPV. – Not measured or not relevant for the affected group. Psychiatric instrument used: ^*a*^Composite Abuse Scale (CAS) (Hegarty et al., 1999). ^*b*^Trauma checklist (Neuner et al., 2004). ^*c*^Things I Have Seen and Heard (TSHS) (Richters and Martinez, 1990). ^*d*^Parental Bonding Instrument (Parker et al., 1979). ^*e*^Adults: Post-traumatic Stress Diagnostic Scale (Foa, 1995). ^*f*^Children: UCLA Post-traumatic Stress Disorder Reaction Index for DSM-IV (Steinberg et al., 2004). ^g^Patient-Health-Questionnaire for teens (PHQ-9) (Richardson et al., 2010). ^*h*^Strength and Difficult Questionnaire (SDQ), conduct problems scale, only children (Goodman et al., 1998). ^*i*^SDQ hyperactivity scale, only children (Goodman et al., 1998). ^*j*^SDQ emotional symptoms scale, only children (Goodman et al., 1998). ^*k*^SDQ peer problems scale, only children (Goodman et al., 1998). ^*l*^Generalized anxiety disorder (GAD-7), only women (Spitzer et al., 2006).*

### Pre- and Postnatal Stress

All São Gonçalo participants were interviewed individually in their homes. Measures of interpersonal violence during pregnancy focused on the periods before, during, and after pregnancy, as described in Radtke et al. (2011). We used two instruments: the Composite Abuse Scale (CAS) (Hegarty et al., 1999, 2005) and the Things I Have Seen and Heard Scale (TSHS) (Richters and Martinez, 1990). CAS measures the degree of IPV through 30 items scaled from 0 (never) to 5 (daily) that together form four dimensions: severe combined abuse, physical abuse, emotional abuse and harassment. An IPV/CAS score >3 was used as cut-off for generating IPV+ and IPV- groups, which previously been shown to represent consistent IPV (Hegarty et al., 2005). Life time exposure scores used the weighted summarization method described in Radtke et al. (2011). TSHS (Richters and Martinez, 1990) contains 20–26 items that measure exposures to community and domestic violence (CDV). Items are presented in a 5-point format ranking from zero (never) to four (many times). Experience of IPV is not included in TSHS, with exception of witnessing parental interpersonal violence. Trauma exposure was measured through a checklist of 16 items (e.g., natural disasters, physical assault, sexual assault) adapted from Neuner et al. (2004). Maternal care was assessed using the Parental Bonding Instrument (Parker et al., 1979).

### Stress-Related Disorders

Post-traumatic stress disorder symptoms in the children was measured using the University of California at Los Angeles Post-traumatic Stress Disorder Reaction Index for DSM-IV (Steinberg et al., 2004). For adults the Post-traumatic Stress Diagnostic Scale (PSSI) was used instead (Foa, 1995). Depression and anxiety were assessed using the Patient-Health-Questionnaire (PHQ-9) (Richardson et al., 2010) and the 7-item scale for generalized anxiety disorders (GAD-7) (Spitzer et al., 2006). Behavioral problems in children was measured using the Strength and Difficult Questionnaire (SDQ), (Goodman et al., 1998) which allocates behavioral attributes to five subscales: emotional symptoms, conduct problems, hyperactivity, peer problems, and prosocial behavior.

### Generating DNA Methylation Data

For the São Gonçalo subjects, saliva samples (2 ml) were collected and DNA isolated using the Oragene-Discover (OGR-500) Collection Kit (DNA Genotek, Ottawa, ON, Canada). DNA was bisulfite-converted and analyzed using HumanMethylation450 BeadChips (Illumina, Inc.) at the Queen Mary University of London (United Kingdom) according to manufacturer’s protocols. Targeted bisulfite resequencing was used to technically validate 10 CpGs on the BeadChips in 33 randomly chosen individuals, using a service provided by Zymo Research Corporation (Irvine, CA, United States). This involved bisulfite conversion using EZ DNA Methylation-Lightning Kit (Zymo), followed by Illumina MiSeq sequencing. Results and primer sequences are found in Supplementary Table S2.

### Preprocessing and Quality Control of DNA Methylation Data

A chart of the full bioinformatics analysis is available in Supplementary Figure S1. Preprocessing and statistical analysis was carried out in R 3.3.2 or SPSS (IBM, v.23). An R script is available in Supplementary Text S1, which automatically performs all preprocessing steps given the input files in Supplementary Data S1–S4. Instructions on how to use the script are contained within the script itself.

The hg19 human reference genome was used in all analysis. Raw São Gonçalo bead array data (idat files) were imported and preprocessed in R using different functions available in minfi package as described in Aryee et al. (2014). Successful bisulfite conversion and hybridization were verified using minfi/qcReport. We used the recommendations by Chen et al. (2013) to filter probes known to cross-hybridize with multiple genomic locations (*n*_probes_ = 30,969), contain or neighboring common polymorphisms (*n*_probes_ = 162,255), or were annotated to the X/Y chromosomes (*n*_probes_ = 8,360). Probes with low detection signals (*p* > 0.01; *n*_probes_ = 5,526) in any of the samples were also excluded (Aryee et al., 2014). The final São Gonçalo dataset contained 272,636 high quality CpGs after filtering.

Confirmation that individuals were assigned to unique families was done using the 65 SNP probes available on the beadchip. We also confirmed that all subjects’ sex, age, and sample type (saliva) matched the predicted epigenetic sex, age and sample type estimated by minfi (Aryee et al., 2014). In total, the São Gonçalo dataset contained 120 children (prenatal IPV+ = 21, IPV- = 99) and 122 adult women (prenatal IPV+ = 23, IPV- = 99), of which most were mothers to the children (pregnancy IPV+ = 18; IPV- = 104; full families = 115).

### Genome-Wide Differential DNA Methylation Analysis

Filtered data were quantile normalized using default settings in minfi/preprocessQuantile. Logit-transformed beta values (*M*-values) were subjected to a robust linear regression model to identify significantly differentially methylated (DM) probes in association with prenatal IPV, adjusted for multiple testing (Benjamini–Hochberg method), using the Limma package (Ritchie et al., 2015). To minimize the effect of outliers due to the presence of rare SNPs in target CpG sites, we used the robust method in the lmFit function in limma (for more details, see Supplementary Figure S4). For the São Gonçalo children and women the model had prenatal IPV exposure (IPV+ versus IPV-) as main effect, and age, biological sex, prenatal CDV, and prenatal trauma scores (maternal exposures during pregnancy) as covariates. Again an IPV/CAS score >3 was used as cut-off for generating prenatal IPV+ and IPV- groups (Hegarty et al., 2005). An extended model with additional covariates is present in Supplementary Table S1. The covariates in the analysis of the contrast datasets may have differed (see section “Analysis of Contrast Datasets” below).

To generate the genomic context graphs of *NR3C1* and *FKBP5* (Supplementary Figure S5), data were downloaded from UCSC genome browser (hg19). Histone and transcription factor binding data were taken from the default Integrated Regulation from ENCODE Track (Layered H3K4Me1, H3K4Me3, H3K27Ac, and Txn Factor ChIP) which contains data from multiple cell lines. For the Txn Factor ChIP, only the areas closest to candidate CpGs were considered, and due to the relative abundance of peaks at the *NR3C1* CpG, only transcription factors with at least two peaks was retained for this gene (links to original graphs see Supplementary Figure S5). To generate heterochromatin/euchromatin coverage graphs (Figure 2D), ENCODE data for PBMC specific H3K9me3 and H3K4me3 ChIP-seq experiments were downloaded from the SYDH Histone Track in the USCS genome browser (GEO access: GSM788075 and GSM788084). To minimize differences in background noise between H3K9me3 and H3K4me3, the *y*-axes of these graphs were set by the average signal of the first and last bp of the 6,000 bp window (±3,000 bp relative CpG location). The same approach, but using genomic ranges downloaded from the ChromHMM Core 15 dataset (Ernst and Kellis, 2012) (Roadmap Epigenomic Project^1^), was used to visualize the overlap with defined chromatin states in cells present in saliva (for details, see Supplementary Figure S6). To partly control for polymorphism acting in trans we searched the mQTLdb database^2^ (accessed 07/15/2018) for previously identified methylation QTLs in the 31 differentially methylated CpGs reported in Table 2 (see footnote 2). The PLINK childhood (age 7) and adolescence (age 15–17) datasets (at any genomic distance) were used since these were corrected for sex, batch effects, cellular heterogeneity and genetic structure.

**Table 2:** Differentially methylated CpGs between São Gonçalo children exposed to prenatal IPV (*n* = 21) and controls (*n* = 99).

Rank	CpGprobe ID	Log_2_fold change	Average % methylation (SEM)	Original*p*-value	FDR adjusted*p*-value	Closestgene	Psychiatricassociation ^c^
1	cg19014730	0.31	70.71 (0.38)	2.60E-07	0.05	*FKBP5*	Binder et al., 2008; Klengel et al., 2013
2	cg04967982 ^a^	–0.35	35.79 (0.59)	4.39E-07	0.05	*OTUD7A*	Egger et al., 2014; Uddin et al., 2018
3	cg11608150 ^b^	–0.82	44.12 (1.61)	5.07E-07	0.05	*VTRNA2-1*	
4	cg12661272	0.16	79.10 (0.19)	1.12E-06	0.07	*STK32C*	Dempster et al., 2014
5	cg02638458	–0.23	26.32 (0.30)	1.27E-06	0.07	*CNPY1*	
6	cg05292788	0.35	86.46 (0.24)	1.52E-06	0.07	*GABBR1*	Fatemi et al., 2011; Vangeel et al., 2017
7	cg24188111	0.20	6.69 (0.10)	1.91E-06	0.07	*DHX29*	
8	cg24137863	0.26	81.33 (0.25)	2.40E-06	0.07		
9	cg11147724	0.19	85.30 (0.17)	2.46E-06	0.07	*PTPRN2*	Jarick et al., 2012
10	cg08857436	0.35	89.36 (0.21)	3.20E-06	0.08	*CDK14*	
11	cg08128826	–0.45	36.43 (0.62)	3.61E-06	0.08	*LHX9*	Yamazaki et al., 2015
12	cg27478707	0.28	82.96 (0.26)	3.87E-06	0.08	*HLA-DMB*	
13	cg17403899	–0.22	72.21 (0.27)	4.45E-06	0.08	*CNFN*	
14	cg21721210	–0.29	20.02 (0.29)	4.79E-06	0.08	*ACP7*	
15	cg04914231	–0.37	27.19 (0.47)	5.16E-06	0.08	*INO80E*	
16	cg07305719	–0.27	82.76 (0.25)	5.31E-06	0.09	*ZNF365*	Ayyappan et al., 2009; Chaste et al., 2015
17	cg04515200 ^b^	–0.70	25.35 (0.88)	5.57E-06	0.09	*VTRNA2-1*	
18	cg00058413	0.27	85.79 (0.26)	5.87E-06	0.09	*DTWD2*	Ellsworth et al., 2013; Pan et al., 2013
19	cg10549227	0.24	85.34 (0.17)	6.29E-06	0.09	*OXT*	Ferguson et al., 2000; Feldman et al., 2016
20	cg20895092	–0.21	81.23 (0.24)	6.43E-06	0.09	*SPDYC*	
21	cg19365706	0.18	91.97 (0.09)	6.51E-06	0.09	*EPHB4*	
22	cg21552292	0.20	79.39 (0.25)	7.60E-06	0.09	*TRIO*	
23	cg10188592	–0.42	4.41 (0.11)	7.62E-06	0.09	*HSPE1*	
24	cg22043788	–0.30	90.71 (0.15)	7.81E-06	0.09	*TBCD*	
25	cg10616337	–0.25	8.30 (0.13)	7.84E-06	0.09	*CRLS1*	
26	cg08108641	–0.22	75.23 (0.29)	9.60E-06	<0.10	*NTNG2*	
27	cg03197792	0.18	85.34 (0.14)	1.05E-05	<0.10	*HDLBP*	Felder et al., 2009
28	cg08205099	0.20	83.80 (0.21)	1.07E-05	<0.10	*DLGAP2*	Marshall et al., 2008; Chertkow-Deutsher et al., 2010
29	cg05291178	0.21	89.47 (0.38)	1.08E-05	<0.10	*FANCD2OS*	
30	cg13494826	0.20	66.89 (0.27)	1.13E-05	<0.10	*PRRX2*	
31	cg00772663	0.32	86.18 (0.27)	1.13E-05	<0.10	*CTNNA2*	Dempster et al., 2011; Elia et al., 2011
…	…	…	…	…	…	*…*	
(39)	(cg18068240)	–0.32	5.44 (0.11)	1.18E-05	0.12	*NR3C1*	McGowan et al., 2009; Spijker and van Rossum, 2012


*Percent methylation was calculated from β-values, while fold changes and statistical analysis were calculated from *M*-values as recommended by Du et al. (2010). FDR indicates *p*-value after correcting for multiple testing using the Benjamini–Hochberg procedure. In total 272,636 CpGs were analyzed. ^*a*^ Possibly affected by transacting polymorphism according mQTLdp/PLINK (http://mqtldb.org/). ^*b*^ Likely affected by loss-of-methylation site polymorphism (see Supplementary Figure S4). ^*c*^ Previous psychiatric associations in the literature was limited to two references.*

### Stress-Induced Methylome Switching and Retrotransposon Analysis

The R script available in Supplementary Text S1 will perform a methylome switching analysis on normalized beta-values from the São Gonçalo samples (Supplementary Data S1), as well as downloaded data from the Grady Study (see section “Analysis of Contrast Datasets” below). Methylome switching was calculated by classifying each CpG into two categories: >50% or <50% methylation (hetero- and eu- chromatin-like, respectively) based on mean percent methylation of each CpG across subjects. We used K-means clustering analysis (*K* = 2), in the children and mothers, respectively, to validate the 50% cut-off. All classification was done within cohorts. As a result, the number of CpGs in each category differed slightly between datasets, since some CpGs may have been classified as heterochromatin-like in one cohort, but euchromatin-like in another. In downstream analysis, we further divided these two categories into those CpGs with or without a repeat region within ±50 bp, using the GenomicRanges package (Carlson et al., 2014) and repeat coordinates from RepeatMasker^3^. Average DNA methylation of each category were then calculated for the top 1% differentially methylated CpGs in respective dataset. We choose to rank the CpGs by Bayesian *B*-values instead of *p*-values, to circumvent any issue with *p*-value cut-off levels. The 1% threshold was chosen based on previous data (Nätt et al., 2015), as well as observations in Figure 2E. The progressive proportion graphs (Figure 2E) were constructed using information from RepeatMasker (repeat class; see footnote 3), and UCSC genome browser (CpG islands and exons). Since CpGs available on the Illumina HumanMethylation450 BeadChips are not a random subset, we choose to normalize the graphs so that all category lines originated from a 50% ratio at the level of all probes (100% top ranked). Bootstrap resampling to generate expected distributions given the CpG panels available on the beadchip was conducted using the boot package in R. Information in RepBase (Bao et al., 2015) was used to extract probes within ±50 bp of recently active L1 retrotransposons unique to the human linage.

### Cell Type and Genetic Ancestry

To control for genetic structure and cell type heterogeneity bias between IPV+ and IPV- groups in São Gonçalo children, we extracted the minfi beta-values, imported them into GLINT 1.0.3 (Rahmani et al., 2017b) and attained the factor scores from the methods listed below. Analysis within GLINT used the default settings. For genetic ancestry analysis EPISTRUCTURE was used (Rahmani et al., 2017a), while ReFACTor was used for reference free cell type analysis (Rahmani et al., 2017b). Since, saliva contains white-blood cells (Theda et al., 2018) we used the Houseman (Houseman et al., 2012) blood reference available in GLINT, as well as a custom-made reference for panel-based cell type analysis. The custom reference was built using the Houseman method (Houseman et al., 2012), but was based on CpGs differentially methylated between saliva, buccal and a pool of the other tissues available in GEO dataset GSE48472. The reference panel can be found in Serpeloni et al. (2017), imported into GLINT and integrated into Houseman pipeline for saliva cell type analysis.

### Statistical Models

For an overview of the statistical models used, see Supplementary Figure S1. Following the initial genome-wide analysis in R/limma, we applied general linear models (GLMs) in SPSS 23 for all group comparisons. Care was taken to assure that the data met GLM assumptions. For the São Gonçalo dataset, model design was the same as for genome-wide analysis, using the same IPV classification (main factor) and covariates (age, sex, prenatal trauma, and prenatal CDV). Models of the contrast datasets are presented below.

### Analysis of Contrast Datasets

If not otherwise stated, sample handling, preprocessing and genome-wide analysis of the contrast datasets were performed the same way as for the São Gonçalo children and women datasets. For consistencies, we only analyzed CpGs that were in common with the São Gonçalo datasets.

Refugee children presented in Figure 3 came from war refugees that migrated to Germany between 2010 and 2016 (*n* = 48; 60% females; 11–19 years old; 75% middle eastern; 83% Islamic). Bisulfite converted saliva DNA was hybridized to Infinium MethylationEPIC (Illumina, Inc.), and was therefore separately analyzed compared to the other contrast datasets. 239,442 CpGs were in common with the São Gonçalo datasets after filtering. To test differences between IPV+ (*n* = 15) and IPV- (*n* = 33) we used a similar statistical model as for the São Gonçalo children, with the same IPV threshold (CAS score > 3). As covariate, instead of prenatal CDV, we used prenatal scores of organized violence calculated using the Event Checklist for War, Detention, and Torture Experiences as described in Onyut et al. (2009). Results were not affected by cell-type heterogeneity and genetic ancestry as described above.

Bisulfite converted DNA from the São Gonçalo women and the Grady datasets were hybridized to the HumanMethylation450 BeadChips (Illumina, Inc.) and was therefore analyzed together, only retaining CpGs that reached the detection signal threshold across all cohorts including the São Gonçalo children (*n*_CpGs_ = 235,960). The São Gonçalo women (mothers) dataset was presented above, with the São Gonçalo children.

The Grady dataset has been described in details previously (Zannas et al., 2015). Files were downloaded as Illumina HumanMethylation450 BeadChips raw signals from Gene Expression Omnibus (GEO, accession number: GSE72680). Only samples with sex, age (co-variates) and information on whether the participant had undergone psychiatric treatment (main factor) were retrained and imported into R. Quality control, filtering and normalization were performed as for the São Gonçalo dataset. The final dataset contained 367 individuals (70% females; 18–77 years old; 91% African American). Groups were generate according to psychiatric treatment of either depression, bipolar disorder, PTSD or anxiety disorder (*n*_yes_ = 140, *n*_no_ = 227). We did not observe group differences in cell-type heterogeneity and genetic ancestry as described above. The R script available in Supplementary Text S1 will automatically download, preprocess, and perform an epigenome-wide association and methylome switching analysis on the Grady data, using the input files in Supplementary Data S1–S4. Instructions are contained within the script.

### Ethics Statement

Approvals were obtained in accordance with the Declaration of Helsinki by the Ethical Committee of the University of Konstanz (Germany; permit UNIKON IRB st No. 4/2015) and the National Commission for Ethics in Research (Brazil; permit CAAE 16375413.1.0000.5240). We obtained written informed consent from adults/parents and written assent from children.

## Results

### Psychiatric Burden in Children Exposed to Prenatal IPV

For an overview of all analysis, see Supplementary Figure S1. In the São Gonçalo samples, 19% of all mothers reported IPV during their pregnancy (*n* = 244; both maternal and grand-maternal generations included). As expected, IPV during pregnancy in the maternal generation (*n* = 122) was positively correlated with exposure to lifetime trauma, and community/domestic violence (CDV) other than IPV, as well as depression, PTSD and anxiety symptoms (Table 1). Children exposed to lifetime trauma, and CDV also showed the expected psychiatric problems, which validated the collection methods. Against previous findings in humans, however, children exposed to prenatal IPV did not present mental and behavioral problems (Table 1). Only a weak relationship with emotional symptoms (e.g., expressing more fears and worries) was present.

**FIGURE 1:**
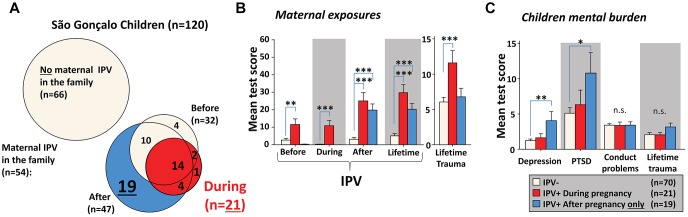
Missing psychiatric problems in São Gonçalo children following prenatal IPV. Most mothers experiencing pregnancy IPV were also exposed to IPV before conception or after giving birth **(A)**. A substantial number of mothers experienced IPV only after birth. Despite similar maternal IPV exposures after birth in both prenatally and only postnatally exposed children, and even higher lifetime trauma exposures in mothers experiencing IPV during pregnancy **(B)**, only children living in families with mothers exposed to IPV only postnatally expressed more depression and PTSD symptoms **(C)**. Results in **(B,C)** originates from Bonferroni corrected *post hoc* tests, using general linear models with age, sex, pregnancy/prenatal trauma and CDV scores as covariates. Error bars indicates ±SEM. ^∗∗∗^*p* < 0.001, ^∗∗^*p* < 0.01, ^∗^*p* < 0.05.

As described by Hegarty et al. (2005), an IPV score of more than 3 in CAS reliably detects IPV exposure. We used this threshold to generate groups that had or had not been exposed to IPV. Only 22% of the mothers that experienced pregnancy IPV had been exposed to prenatal IPV themselves, confirming the low correlation between pregnancy and prenatal exposures reported in Table 1. Pregnancy IPV in the São Gonçalo mothers often coincided with IPV before and/or after pregnancy (Figure 1A). Several children, however, had mothers that experienced IPV exclusively after birth. These children had similar load of maternal IPV after birth, and their mothers experienced similar lifetime IPV exposure, as children that experienced maternal IPV prenatally (Figure 1B). Nevertheless, only children experiencing maternal IPV exclusively after birth expressed elevated levels of mental symptoms compared to controls (Figure 1C). This implies that prenatal IPV in the São Gonçalo children may have led to some sort of resilience against experiencing/witnessing maternal IPV.

### Prenatal IPV Affects Genes Relevant for Stress and Psychiatric Disorder

We next explored epigenetic biomarkers by measuring genome-wide saliva DNA methylation using Illumina HumanMethylation450 BeadChips (children = 120; women = 122; mother/children family pairs = 115). To validate the arrays, we technically replicated 10 CpGs covered by the BeadChip in 33 randomly chosen individuals (children = 18; women = 15) using targeted bisulfite resequencing, which showed strong inter-assay/subject reliability (subject mean *r*^2^ = 0.94; max = 0.96; min = 0.90; each subject had *p* < 0.001; Supplementary Table S2). Furthermore, we applied established practices to exclude CpGs with common polymorphisms and without reliable detection signals in all samples (see section “Materials and Methods”). Since the children samples contained both males and females, we also excluded the sex chromosomes, resulting in a final dataset of 272,636 CpGs.

**FIGURE 2:**
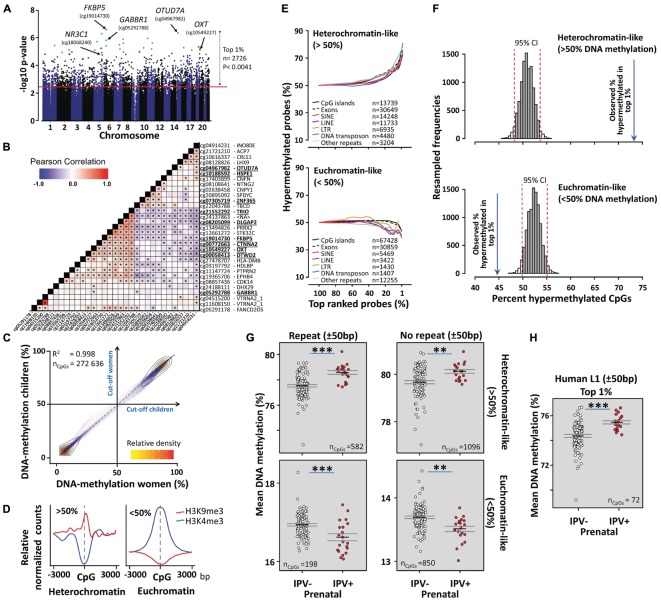
A methylome switch in the São Gonçalo children. **(A)** Manhattan plot showing the top 1% differentially methylated CpGs (Supplementary Data S1) in the São Gonçalo children exposed to prenatal IPV (IPV+) compared to controls (IPV–). Green dots show the significant CpGs from Table 2. **(B)** Differentially methylated CpGs in São Gonçalo children correlated across subjects. Asterisks here represent at least *p* < 0.05 significant Pearson correlations between target CpGs. **(C)** DNA methylation strongly correlated between São Gonçalo children and women. Small blue dots show average DNA methylation of each analyzed CpG in children and women, respectively (*n* = 272,636). A 50% cut-off in DNA methylation efficiently caught two distinct clusters verified with K-means cluster analysis (see main text). **(D)** The >50% DNA methylation cluster associated with the H3K9me3 heterochromatin marker, while the <50% cluster associated with the H3K4me3 euchromatin marker, as measured in previous ChIP-seq experiments of white blood cells. **(E)** Line graphs showing an accumulation of hypermethylated CpGs in heterochromatin-like regions of the top most differentially methylated CpGs between prenatal IPV+ and IPV– children (top graph). This was accompanied by a reduction of hypermethylated CpGs in euchromatin-like regions (lower graph). CpGs were categorized by being within ±50 bp of repetitive genomic features (SINE, LINE, LTR, DNA transposon, other repeats) or gene associated features (exons and/or CpG islands; no repeat within ±50 bp). **(F)** Comparing the observed number of hypermethylated CpGs in the top 1% differentially methylated CpGs with confidence intervals of the expected distributions generated by resampling 1% randomly from all analyzed CpGs (10,000 resamples), confirmed that the prenatal IPV+ children had more hypermethylated CpGs in heterochromatin-like regions, and less hypermethylated CpGs in euchromatin-like regions, compared to controls. **(G)** Averaging the top 1% CpGs into either heterochromatin-like CpGs with or without repeats (±50 bp), and euchromatin-like CpGs with or without repeats, showed that the effects were more pronounced in repetitive regions. **(H)** Prenatal IPV also led to more methylation in CpGs within ±50 bp of recently active retrotransposons. Error bars indicates ±SEM. Asterisks in **(G,H)** represent results from general linear models with age, sex, prenatal CDV, and prenatal trauma scores as covariates. ^∗∗∗^*p* < 0.001, ^∗∗^*p* < 0.01.

After correcting for multiple testing using the Benjamini–Hochberg procedure, 31 CpGs (Table 2 and Figure 2A) were differentially methylated between children exposed to prenatal IPV (IPV+, *n* = 21, mean age = 13.4 ± 0.5) and controls (IPV-, *n* = 99, mean age = 13.7 ± 0.3 SEM) using the Hegarty threshold described above (Hegarty et al., 2005) and a false discovery adjusted *p*-value < 0.1. To increase specificity toward IPV, the analysis was corrected for the covariates sex and age, as well as maternal exposures to pregnancy trauma and other types of CDV. Supplementary Table S1 shows a comparison between this model and an extended model including several more possible confounds like cell-type heterogeneity, genetic structure, family income and maternal smoking/alcohol use during pregnancy, which showed limited effects on the results. Direct between group comparisons of cell-type heterogeneity (Supplementary Figure S2), genetic ancestry (Supplementary Figure S2), reported age/epigenetic age (Supplementary Figure S3), and other possible confounds (Table 1) confirmed that these variables had limited effects on the results. We also applied a statistical strategy called robust statistics, to limit loss-of-methylation site polymorphism bias between the prenatal IPV+ and IPV- groups (for more information, see Supplementary Figure S4). To partly control for distant polymorphisms affecting DNA methylation, we searched the mQTLdb/PLINK database (see footnote 2) for previously identified methylation QTLs affecting these 31 CpGs. Only one of the 31 CpGs, cg04967982, had previously been associated with *trans*-acting polymorphism in juvenile/adolescence samples, suggesting limited effects of this type of genomic interaction.

Ranked 1 of 272,636 CpGs analyzed was a CpG in the gene body of *FKBP5* (Table 2 and Supplementary Figure S4), which codes for FKBP51, the chaperone protein for GR which was presented in the text above. Also, a CpG in the promoter of the gene coding for GR, *NR3C1*, was top ranked (rank 39; Table 2). In both genes, the significant CpGs were in regions previously characterized by histone H3K27 acetylation and H3K4 mono/tri-methylation, which are histone marks commonly associated with active transcription (Supplementary Figure S5). While DNA methylation at the *NR3C1* CpG supported an active promoter, by being poorly methylated, *FKBP5* showed higher levels of DNA methylation, suggesting local silencing (Table 2). The *FKBP5* CpG, however, showed substantial variation, and has a plausible binding site for the activator protein 1 (AP1) complex: a potent activator of gene transcription following dimerization of c-Fos and c-Jun (Supplementary Figure S5). A qualitative guess is therefore that the *FKBP5* site is located within an enhancer region, but more evidence is needed.

Many other differentially methylated CpGs were located in or close to genes previously associated with stress and psychiatric disorders, such as the genes for oxytocin (*OXT*) and DLG Associated Protein 2 (*DLGAP2*) that have commonly been associated with deficits in social behavior and autism spectrum disorder (Marshall et al., 2008; Feldman et al., 2016). Furthermore, a subunit for GABA receptor 1B (*GABBR1*), DTW Domain Containing 2 (*DTWD2*), and DISC1-Binding Zinc-Finger Protein (*ZNF365*), have been associated with multiple often comorbid disorders, including addiction, bipolar disorder, and schizophrenia (Ayyappan et al., 2009; Fatemi et al., 2011; Pan et al., 2013; Augier et al., 2018). Of interest for our study, polymorphism in *DTWD2* has shown to interact with *FKBP5* in *trans* (Ellsworth et al., 2013) and *GABBR1* has been associated with DNA methylation changes in newborns following maternal anxiety during pregnancy (Vangeel et al., 2017). Furthermore, the high number of psychiatrically relevant genes may indicate co-regulation involving, for example, common transcription factors. This was consistent with high inter-subject correlations across many of the differentially methylated sites (Figure 2B).

Since the maternal generation was also assessed for genome-wide DNA methylation, we cross-examined the children results in their mothers. No correlations within families (*n* = 115 mother/child pairs) were found for the 31 significant CpGs identified in the children, providing evidence against either genetic or intergenerational epigenetic inheritance. Changes in the children following prenatal IPV were therefore likely independent of the changes following the same exposures in their mothers.

### Stress Tolerance and Methylome Switching in São Gonçalo Children

Similar changes in *FKBP5* and *NR3C1* DNA methylation reported by others (Binder et al., 2008; McGowan et al., 2009; Spijker and van Rossum, 2012; Klengel et al., 2013; Zannas and Binder, 2014) suggest that prenatal IPV exposed São Gonçalo children would have higher availability of GR, resulting in enhanced negative feedback of the HPA-axis, and therefore faster stress recovery than controls. While this may explain why these children seemed protected from the psychiatric consequences of prenatal IPV, only studying brain receptor function may fully addressed this hypothesis. Considering the challenges to study the São Gonçalo population this was out of our scope. Furthermore, while DNA methylation in *NR3C1* and *FKBP5* have been suggested to be indicators of mental burden, inconsistencies between studies have questioned their biomarker potential (Palma-Gudiel et al., 2015; Argentieri et al., 2017). Therefore, we sought to explore epigenetic indicators of stress tolerance that are not dependent on single CpGs, but instead averages information across genomes. Analyzing our data this way, would make our observation more robust against technical noise and decrease the risk of making false discoveries due to multiple testing.

Exposures to stress, aging and disease have been shown to associate with genome-wide losses of DNA methylation in heterochromatin-like genomic regions rich in DNA methylation (Jones et al., 2015; Nätt et al., 2015; Nätt and Thorsell, 2016). More DNA methylation in heterochromatin-like regions would therefore indicate stress/disease resilience and deaccelerated aging. Based on this, we simply categorized all analyzed CpGs into hyper (>50%) or hypo- (<50%) methylated (Figure 2C). The seemingly arbitrary 50% cut-off overlapped to >98.7% with two clusters, independently replicated in the São Gonçalo children and women, and unbiasedly generated using K-means clustering analysis (*K* = 2). Clusters were also inversely associated with the heterochromatin marker H3K9me3, and euchromatin marker H3K4me3, as previously measured in mono nuclear leucocytes (Figure 2D). Inversed chromatin states of these clusters were also confirmed in relevant cell types using ChromHMM data downloaded from the Roadmap Epigenomics Project, which reports chromatin states based on machine learning of five different chromatin markers (Supplementary Figure S6).

Exploring the hetero- and eu-chromatin-like clusters revealed a never described phenomena following prenatal stress in humans. In heterochromatin-like regions, the number of hypermethylated CpGs in prenatal IPV exposed children compared to controls progressively increased the closer to the top most differentially methylated CpGs that was targeted (top graph Figure 2E). The gain in DNA methylation of heterochromatin-like regions was accompanied by a rebound in euchromatin-like regions (bottom graph Figure 2E). This resulted in what appeared to be a methylome switch, where prenatal IPV exposure led to more methylation in heterochromatin-like and less methylation in euchromatin-like regions of the top regions affected by prenatal IPV. The switch affected many types of genomic regions but was most pronounced in several classes of transposons and other repetitive genomic elements (Figure 2E). Poorly methylated exons and CpG islands depleted of repetitive regions appeared slightly protected against the switch, at least in euchromatin-like regions.

Given the explorative findings in Figure 2E, we looked more closely at the 1% top differentially methylated CpGs between IPV+ and IPV- children (Supplementary Data S1). The proportion of hypermethylated regions in IPV+ children was far greater than what was expected by confidence intervals generated using 10,000 bootstrap resamples of the original 272,636 analyzed CpGs post filtering (top graph Figure 2F). While not as pronounced, the opposite relationship was observed in the eu-chromatin like regions (bottom graph Figure 2F). This confirms statistically the overrepresentation observed in Figure 2E, and that the switch was not biased by the CpG panel chosen for the Illumina HumanMethylation450 BeadChip.

A methylome switch was also present when averaging the DNA methylation levels over heterochromatin-like repetitive and non-repetitive, as well as euchromatin-like repetitive and non-repetitive regions (Figure 2G). Again, the most pronounced effect was found in repetitive regions.

Stress and age-related loss of DNA methylation in repetitive heterochromatin-like regions may partly involve a mechanism that reactivates intact retrotransposons (Wood and Helfand, 2013; Gorbunova et al., 2014; Nätt and Thorsell, 2016). These virus-like elements are often found in repetitive genomic regions and are normally silenced by DNA methylation. It is believed that when this epigenetic control mechanism is compromised, these elements may reactivate, spread to other genomic locations, leading to disruption of genome integrity, and increased nuclear instability (Nätt and Thorsell, 2016). Since the strongest effect by prenatal IPV was found within repetitive heterochromatin-like regions (Figure 2E–G), we therefore limited our analysis to only involve known recently active L1 retrotransposons specific to the human linage. As expected, prenatal IPV exposed São Gonçalo children showed more DNA methylation in these regions, suggesting that these children may have stronger protection against nuclear instability caused by reactivation of retrotransposons compared to the controls (Figure 2H).

### Children Exposed to Both Prenatal IPV and Postnatal Violence Share a Unique Relationship

To put the findings in the São Gonçalo children into a wider context, we compared the results with three other datasets relevant for prenatal IPV and psychiatric burden.

**FIGURE 3:**
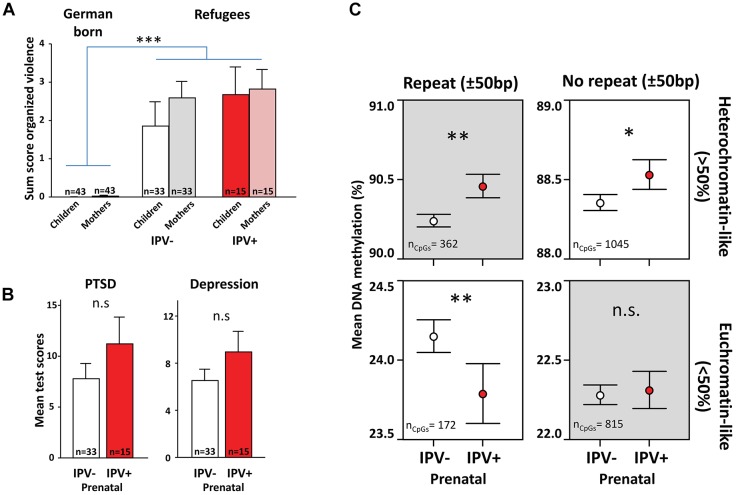
A methylome switch in prenatally stressed war-exposed refugees. Graphs show results from an independent study in refugees primarily born in countries of the Middle East which migrated to Germany between 2010 and 2016. **(A)** Refugee mothers and children had higher life-time exposure to violence than reference groups born in Germany. Community violence scores used for the São Gonçalo samples was not available here. Instead graphs indicate organized violence scored using the Event Checklist for War, Detention, and Torture Experiences, as described in Onyut et al. (2009). **(B)** Using the same methods for scoring symptoms of PTSD and depression as were used for the São Gonçalo samples, refugee children that experienced prenatal IPV (IPV+) did not show more symptoms of PTSD and depression compared to controls (IPV–). **(C)** Just like the São Gonçalo children, refugee children that experienced prenatal IPV had more DNA methylation in heterochromatin-like repetitive regions than controls, accompanied with what appeared to be a rebound in euchromatin-like repetitive regions. Analysis was done on CpGs in common post filter between refugees and São Gonçalo cohorts (*n* = 239,442). General linear models were used with age, sex and prenatal exposure to trauma and organized violence as covariates. ^∗∗∗^*p* < 0.001, ^∗∗^*p* < 0.01, ^∗^*p* < 0.05.

Firstly, we tried to validate the main analysis in saliva samples from an independent juvenile cohort of war immigrants with primarily Middle Eastern origin (IPV+ = 15, mean age = 14.7 ± 0.6; IPV- = 33, mean age = 15.4 ± 0.4 SEM). This cohort had also experienced high levels of violence both in the maternal and juvenile generations (Figure 3A). While the refugee children did not present as pronounced resilience in their psychiatric profiles following prenatal IPV as their São Gonçalo counterparts (Figure 3B), the data still showed a similar methylome switch with the strongest effect observed in repetitive heterochromatin-like regions, thus replicating the Brazilian results despite the limited sample size (Figure 3C).

**FIGURE 4:**
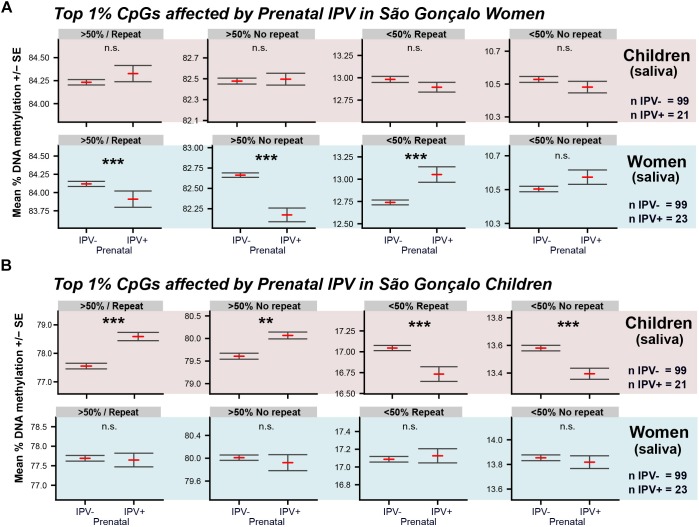
Comparison of the methylome switches in São Gonçalo children and women. Graphs show the methylome switch analyses of the top 1% differentially methylated CpGs affected by prenatal IPV in **(A)** saliva of São Gonçalo children, where the maternal generation was exposed to pregnancy IPV, and **(B)** in saliva of São Gonçalo women, where the grand-maternal generation was exposed to pregnancy IPV. Analyses were done using CpGs in common across all datasets (*n* = 235,960). General linear models were used with age, sex and prenatal exposure to trauma and community violence as covariates. ^∗∗∗^*p* < 0.001, ^∗∗^*p* < 0.01.

Secondly, we used the material collected for the São Gonçalo maternal generation, and investigated prenatal IPV in adult São Gonçalo women, where the grand-maternal generation was exposed to IPV during pregnancy (IPV+ = 23, mean age = 37.5 ± 1.3; IPV- = 99, mean age = 38.3 ± 0.6 SEM). Surprisingly, women in this population showed the opposite methylome switch (Figure 4A) compared to the São Gonçalo children (Figure 4B) following prenatal IPV. These inversed relationships were, however, only present when the analysis was done on the 1% top differentially methylated CpGs identified within each cohort. Thus, São Gonçalo children did not express a methylome switch on the CpGs affected by prenatal stress in adult women, and *vice versa*. This result indicates that prenatal IPV may have unique epigenome-wide consequences across lifespan.

Thirdly, to explore how a methylome switch may appear in subjects with confirmed psychiatric burden we acquired a freely available subset of the Grady dataset (Zannas et al., 2015). This dataset contains 367 individuals, primarily of African-American origin, with white blood cell DNA methylation profiles and information on whether the participants have undergone treatment for psychiatric disorders or not (for more information, see Supplementary Figure S7). We motivated a comparison between blood and saliva with a high density of white blood cells previously observed in saliva (Theda et al., 2018), something that was confirmed in our samples (Supplementary Figure S2). We hypothesized that if a methylome switch truly is a suggestive biomarker for stress exposure and psychiatric vulnerability, patients with a documented history of psychiatric disorders (*n*_yes_ = 140, *n*_no_ = 227) should show the opposite relationship compared to the São Gonçalo children. As hypothesized, psychiatric burden in the Grady cohort showed a similar methylome switch as the São Gonçalo women (Supplementary Figure S7), which was the inverted relationship compared to the São Gonçalo children (Figure 2G, 4B), and the refugee replication cohort (Figure 3C). For regenerating the complete Grady/São Gonçalo comparison, we provide an automated R script in Supplementary Text S1.

Importantly, as indicated by the women/children comparison in Figure 4, the methylome switch observed across the different cohorts were induced at independent genomic sites, where each cohort generated highly unique top 1% subsets with very few overlaps across cohorts (Supplementary Figure S8). In fact, none of the differentially methylated CpGs following prenatal IPV in the São Gonçalo children (Table 1) where among the top 1% differentially methylated CpGs in the other cohorts. This confirms what others have indicated, that single CpGs are relatively poor biomarkers for stress and psychiatric burden (Palma-Gudiel et al., 2015; Argentieri et al., 2017).

## Discussion

We provide both psychiatric and molecular evidence that prenatal stress in humans may have different consequences on mental health depending on the postnatal environment. Children living in the high violence communities of São Gonçalo seemed more resilient to the epigenome-wide and psychiatric consequences of prenatal IPV reported repeatedly by others (Lupien et al., 2009; Fine et al., 2014; Babenko et al., 2015). We found similar effects in an independent sample of children exposed to war violence. Furthermore, consistent with prenatal IPV exposed São Gonçalo children showing more resilience, these children presented the opposite relationships in their epigenetic profiles compared to United States patients with documented history of psychiatric disorders. Together these effects involved hundreds of subjects and thousands of methylation sites, where a clear impact was found in two well established stress response genes, *FKBP5* and *NR3C1.*

The *FKBP5* and *NR3C1* findings are consistent with a reprogramming of the HPA-axis, resulting in an enhanced negative feedback and a faster stress recovery in prenatal IPV exposed São Gonçalo children (Zannas and Binder, 2014). This interpretation should be made with caution, firstly since HPA axis feedback is primarily mediated at the levels of the pituitary, hypothalamus and hippocampus, and secondly since these candidate CpGs are poorly characterized in terms of mechanism. Support for this interpretation is, however, found in our study of peripheral cells populations. Specifically, São Gonçalo children whose mothers were exposed to IPV during pregnancy had more DNA methylation in retrotransposons and heterochromatin-like regions, profiles that previously have been associated with slower aging and lower risk of disease (Wood and Helfand, 2013; Gorbunova et al., 2014; Jones et al., 2015; Nätt and Thorsell, 2016). Chronic cortisol exposures in children (Nätt et al., 2015) and endogenous hypercortisolism (Glad et al., 2017), also associates with similar changes in DNA methylation, which links our findings more directly with stress physiology. It must be noticed, however, that while most studies have reported a relatively linear relationship between stress exposure and age acceleration, one recent finding shows that war-veterans with PTSD can show epigenetic age-deceleration (Verhoeven et al., 2018). Thus, under some circumstances, what appears to be resilience on a molecular level may be associated with a psychiatric disorder on the macro level, which may indicate a non-linear relationship.

If interactions between the prenatal and postnatal environments are important for shaping mental resilience, this may explain the absence of stress-related mental illness in other prenatal stress studies targeting juvenile populations exposed to violence (Roth et al., 2014) or other types of postnatal stress (Sandman et al., 2012). It must, however, be emphasized that although we believe we have one of the first and largest epigenome-wide studies targeting cohorts with consistently high exposures to prenatal IPV, defined as a Hegarty IPV score >3 (Hegarty et al., 2005), each individual cohort represented only a limited amount of prenatal IPV exposed cases. As such, our study should be considered a pilot study for a much larger study involving thousands of subjects, differing on their exposures to prenatal and postnatal violence. Given the available evidence and without acknowledging possible molecular mechanism, we propose three models that should be further investigated in such future research.

*Programmed epigenetic resilience* suggests that the effects of prenatal IPV always turn from being advantageous in childhood to becoming disadvantageous in adulthood. This would explain the inversed relationships seen between São Gonçalo women and São Gonçalo children living in the same environments, but will have a hard time explaining the differences with previous research (Lupien et al., 2009; Fine et al., 2014; Babenko et al., 2015).

*Temporal environmental resilience* suggests that the São Gonçalo communities have changed dramatically during the last decades, thus the environmental factors that shape present day youth were not present when the adult women were in the same age.

The third model we call, *Dynamic environmental resilience*. Here, the present-day São Gonçalo communities must be viewed as a composite of juvenile and adult subcultures, which clearly separates the generations and provide opposite environmental feedbacks to the epigenome. According to previous research (Fine et al., 2014; Babenko et al., 2015), prenatal stress associates with more emotionally impulsive behavior and less response to social cues (including suffering of others), which likely are advantageous traits in juvenile subcultures influenced by gang mentality. Our results indicate that prenatal stress may epigenetically affect emotional behavior (Table 1) and genes controlling social behavior, such as the oxytocin gene (Table 2). Social and emotional problems may, however, be disadvantageous in more societally accepted adult and juvenile subcultures (e.g., work environments), characterized by needs for adhering to social norms, following through with tasks and reacting emotionally to the plight of others. Thus, in this model prenatal stress originally induces the same early life phenotypes across populations (e.g., deficits in social and emotional behavior), which later may be shaped into problems by feedbacks from the postnatal environment (e.g., bullying and social exclusion). While we think this model holds promises for a novel syntheses in molecular psychology, it is in desperate need of further support.

We did not observe any difference in exposures to maternal behavior between prenatal IPV exposed children and controls (Table 1). While our genetic structure analysis (EPISTRUCTURE) partly controls for genetic paternal effects, the effect of the father is a possible confound that also needs to be better addressed in future studies. That the paternal contribution would explain the suggestive effects on psychiatric resilience in the São Gonçalo children is, however, a controversial idea that conflicts with the consensus within child/adolescence psychiatry. This would imply that the fathers, which often are the perpetrators in IPV, may have (epi)genetic benefits in relation to fathers of controls.

We are aware that our results may be viewed as evidence for the Predictive Adaptive Hypothesis (PAR) (Bateson et al., 2014), suggesting that the prenatal environment, experienced by the mother, prepares/adapts the fetus for postnatal life. While we acknowledge this possibility, we recommend caution here. Firstly, our data were collected in humans and therefore are correlational in nature. Secondly, we have no evidence that our observations are adaptive over a life cycle. Thirdly, due to the complexity of human social behavior, subcultures that promote behavioral phenotypes generated by suboptimal brain development (maladaptive traits) are likely to occur. As outlined by the *Dynamic environmental resilience* model, the violent juvenile subcultures that we studied here may represent such a niche.

CpGs previously associated with stress and psychiatric disorders, including CpGs in *NR3C1* and *FKBP5*, replicates poorly across studies (Palma-Gudiel et al., 2015; Argentieri et al., 2017) and have so far failed to translate into reliable clinical tests. Our result confirms this. We have here presented a novel marker that appears to associate with mental burden, namely the stress-induced methylome switch. Fully developed this marker may provide a more robust alternative to candidate CpGs. Like the epigenetic clock (Horvath, 2013), the methylome switch relies on broad epigenome signatures, making it less affected by populational structure and measurement artifacts, such as fluctuations in cell-type heterogeneity. Used properly this marker allow for epigenome studies in relation to more stochastic models of stress and aging, such as the allostatic load (McEwen and Stellar, 1993) and epigenetic drift (Jones et al., 2015) concepts. In such models, it is expected that broad genomic mechanisms, such as a loss of genome integrity, can disassociate the impact of stress at single CpGs (Nätt and Thorsell, 2016). While our methylome switch marker is robust for this kind of stochasticity, it instead suffers from a small effect size. A successful biomarker, however, should primarily rely on the power to detect a true difference. Whether it shows a 0.5% or 50% effect size does not matter given that precision is high. The precision of the methylome switch is high since it summarizes hundreds of measurements at individual CpGs. Future studies must therefore aim to evaluate and refine our suggestive biomarker, rather than dismiss it for being biologically irrelevant due to a low effect size.

## Conclusion

We provide human evidence that children exposed to prenatal stress may experience resilience driven by epigenome-wide interactions. This plausibly translates animal findings and suggests that prenatal psychiatric risk factors may have different, and maybe even opposite, consequences in different world populations. Our findings therefore call for more research on personalizing psychiatric practices according to social and community backgrounds. By studying the gene–environment interactions in some of these world populations, we have also discovered a novel phenomenon that may be refined into a biomarker for mental burden, namely the stress-induced methylome switch. We hope that this discovery may lead to innovative molecular diagnostic tools in psychiatry, with profound impact on our understanding and treatment of mental disorders.

## Author Contributions

TE, DN, FS, MS, and SA: conceptualization. FS, JS, and KR: investigation/sample collection. TE, DN, and SA: supervision. DN, FS, and KR: formal analysis. TE, DN, FS, and TH: methodology. JS and VV: validation. TE, DN, and FS: funding acquisition and writing – original draft preparation. TE, DN, SA, and VV: resources. All authors reviewed and/or edited the manuscript.

## Conflict of Interest Statement

The authors declare that the research was conducted in the absence of any commercial or financial relationships that could be construed as a potential conflict of interest.

## Footnotes

1 https://egg2.wustl.edu/roadmap/web_portal/chr_state_learning.html

2 http://www.mqtldb.org/

3 http://www.repeatmasker.org

## Supplementary Material

Datasheet 1.pdf contains Supplementary Figures and Supplementary Tables.

Datasheet 2.zip contains the script and data for reproducing some of the analysis of this paper, this includes: Supplementary Text S1 which contains the R script that will reproduce our analysis using Supplementary Data S1–S4; Supplementary Text S2 explains the column names in Supplementary Data S1; Supplementary Data S1 is the result table with beta-values for the top 1 % most differentially methylated CpGs between IPV+ and IPV- São Gonçalo children and women; Supplementary Data S2 contains the factors and co-variates for São Gonçalo children and women; Supplementary Data S3 contains the factors and co-variates for the Grady data downloaded from Gene Expression Omnibus; Supplementary Data S4 contains a list of excluded CpGs/probes as described in the Materials and Methods. For possible updates to the R script in Supplementary Text S1 check https://github.com/Danis102 or contact the corresponding author.
