# Supplementary material for: Does Prenatal Stress Shape Postnatal Resilience? – An Epigenome-Wide Study on Violence and Mental Health in Humans
Source: Front Genet. 2019 Apr 16;10:269. doi: 10.3389/fgene.2019.00269 (PMC6477038; doi:10.3389/fgene.2019.00269)
Supplement: Supplementary file 1 — Datasheet 1.pdf contains Supplementary Figures and Supplementary Tables. [file Data_Sheet_1.pdf]

Supplementary Figures and Tables

Does prenatal stress shape postnatal resilience? – An epigenome-wide study on violence and mental health in humans.

Authors: Fernanda Serpeloni, Karl M. Radtke, Tobias Hecker, Johanna Sill, Vanja Vukojevic, Simone Assis, Maggie Schauer, Thomas Elbert, Daniel Nätt

This supplementary includes:

Fig S1 to S8	This PDF page 2-10.
Table S1 to S2	This PDF page 11-12.
Text S1	Separate R script in attached zip file.
Text S2	Separate explanation file in attached zip file
Data S1 to S4	Separate tables in attached zip file.

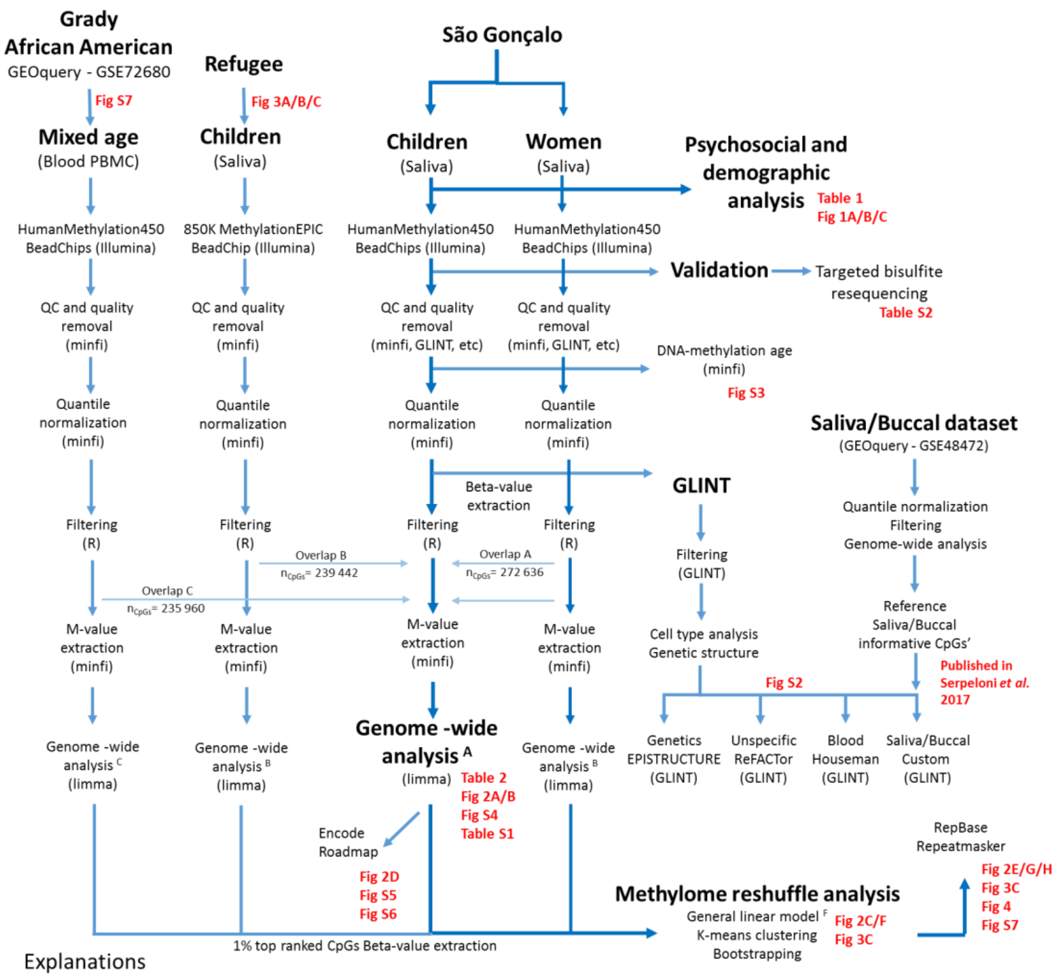

Explanations

R = R project statistical computing (https://www.r-project.org/).
Bioconductor = Open source software for bioinformatics (https://www.bioconductor.org/).
minfi = Minfi package R/Bioconductor (https://bioconductor.org/packages/release/bioc/html/minfi.html).
Limma = limma package R/Bioconductor (https://bioconductor.org/packages/release/bioc/html/limma.html).
GEOquery = GEOquery package R/Bioconductor (http://bioconductor.org/packages/release/bioc/html/GEOquery.html).
GLINT = Software containing the EPISTRUCTURE, ReFACTor, and Houseman methods (http://glint-epigenetics.readthedocs.io/en/latest/index.html)
Custom = Saliva quality control GSE48472
WebGestalt = WEB-based Gene Set Analysis Toolkit (http://webgestalt.org).
Encode = Histone marks and transcription factor ChIP-seq data downloaded from ENCODE: https://www.encodeproject.org/; GSM78807 and GSM788079.
Roadmap = ChromHMM Core 15 dataset from Roadmap Epigenomic Project: https://egg2.wustl.edu/roadmap/web_portal/chr_state_learning.html.
RepBase = Repetitative DNA database (http://www.girinst.org/repbase/).
RepeatMasker = Software detecting repetitive and low complex sequences (http://www.repeatmasker.org/).
K-means clustering = Base R/Bioconductor; used to validate 50% cutoff.
Bootstrapping = Boot package R/Bioconductor; used to validate reshuffling.
Meth = Methylation at specific CpGs using the Illumina HumanMethylation450 or MethylationEPIC BeadChip.
P-IPV = Exposure to maternal prenatal intimate partner violence (children).
Psych = A history of treatment for psychiatric disorder.
Sex = Gender (co-variate in São Gonçalo and German children cohort).
Age = Age according to participant (co-variate in all statistical models).
CDV = Community domestic violence (co-variate to separate effects of IPV from CDV)
Trauma = Exposure to trauma (co-variate to separate effects of IPV from other traumatic events).

Filtering

Overlap A = CpGs in common between São Gonçalo samples post filtering (both women and children).
Overlap B = CpGs in common between São Gonçalo samples and Refugee samples post filtering.
Overlap C = CpGs in common between all cohorts post filtering.

Statistical models (Bold indicates main factor of interest)

[A] Meth=**P-IPV** + Age + Sex + prenatal CDV + prenatal Trauma
[B] Meth=**P-IPV** + Age + prenatal CDV + prenatal Trauma
[C] Meth=**Psych** + Age + Trauma
[F] Same as for Genome-wide analysis for each respective cohort.

For results using an extended model that includes more co-variates, see Table S1.

Fig S1. *Flow chart of all analyses in the experiment.*

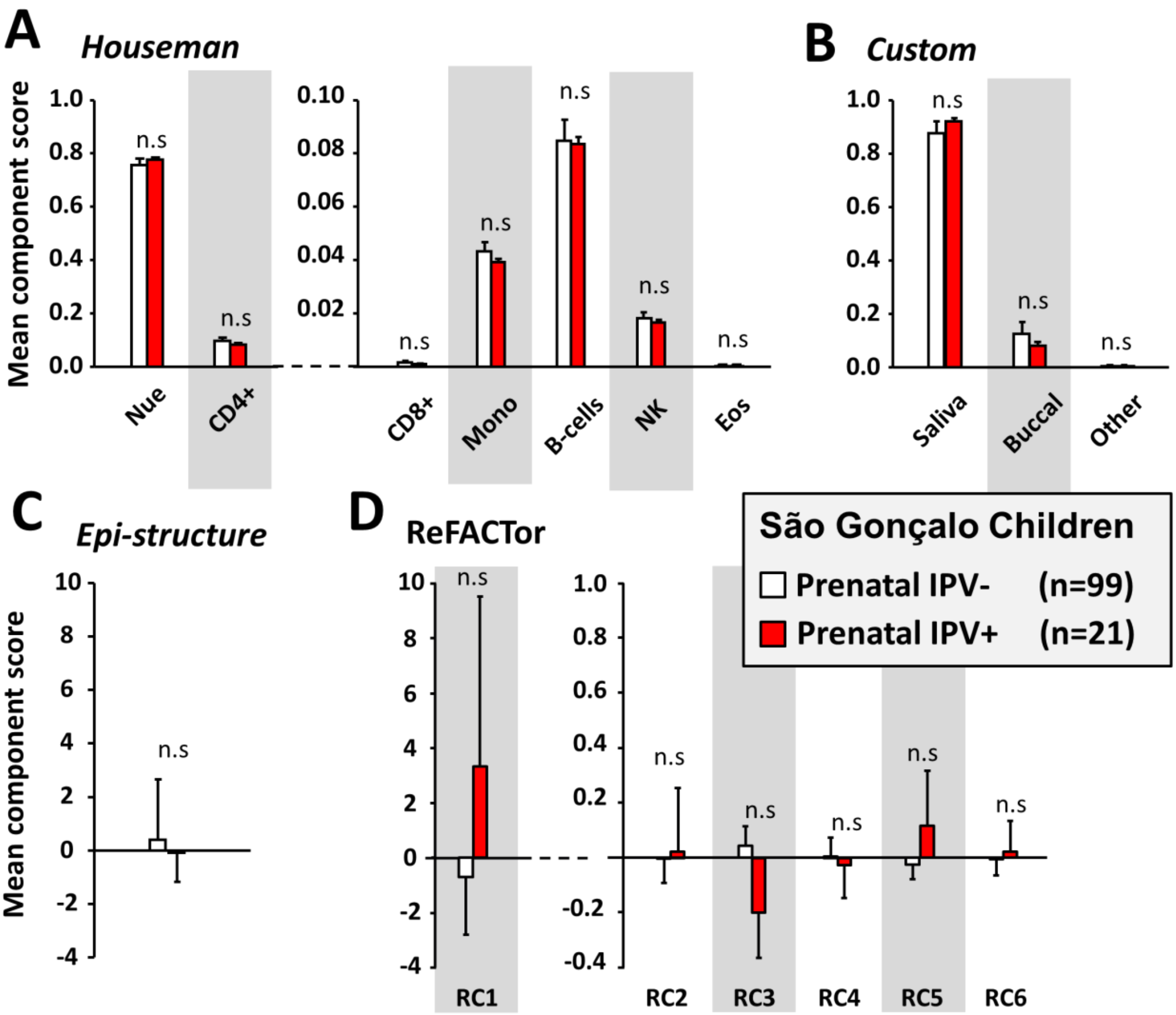

Fig S2. *No difference in cell-type heterogeneity and genetic ancestry between IPV groups in São Gonçalo children.* Graphs show the possible confounding effect of differing cell-type ratios and genetic structure between IPV- and IPV+ groups. Confounds were estimated using the DNA-methylation status at specific CpG panels known to be affected by the specific confound, using validated multivariate models. For more details about these models see methods. Cell-type heterogeneity, measured by the Houseman method with the blood reference panel **(A)** and our custom saliva/buccal reference panel **(B),** was not affect by prenatal IPV exposure in the São Gonçalo children. Neither were the groups differentially affected by genetic ancestry measured by the EPISTRUCTURE method **(C)**. Similarly, no difference between IPV groups was observed using the independent ReFACTor method **(D)**. ReFACTor scores gives cell-type ratios without the need for a unique cell-type reference panel. Bars represent the factor scores extracted from the GLINT software after the co-variants sex, age, prenatal CDV and trauma exposure have been adjusted for. n.s. indicates a non-significant result between IPV- and IPV+ using a Mann-Whitney U tests (not corrected for multiple testing). Error bars indicates SEM.

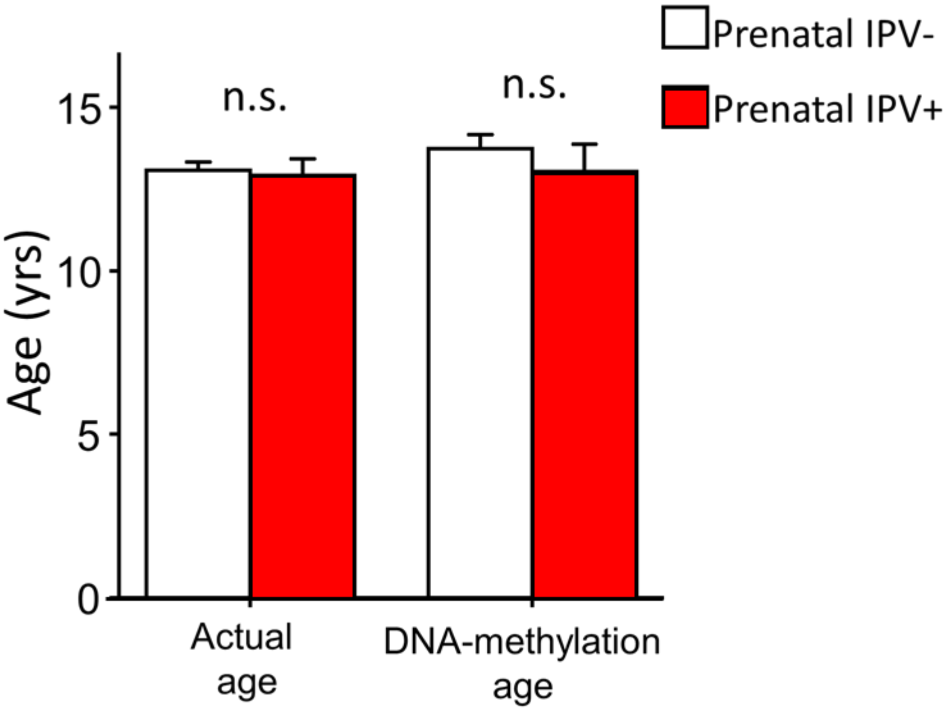

Fig S3. *Epigenetic age was not affected by* prenatal *IPV*. Neither actual age nor DNA-methylation age, as calculated using the Hovarth method (see Methods for details), were associated with exposure to prenatal IPV in the São Gonçalo children. Nevertheless, we included reported age as a covariate in all our analysis (IPV– n=99, IPV+ n= 21; error bars indicate SEM).

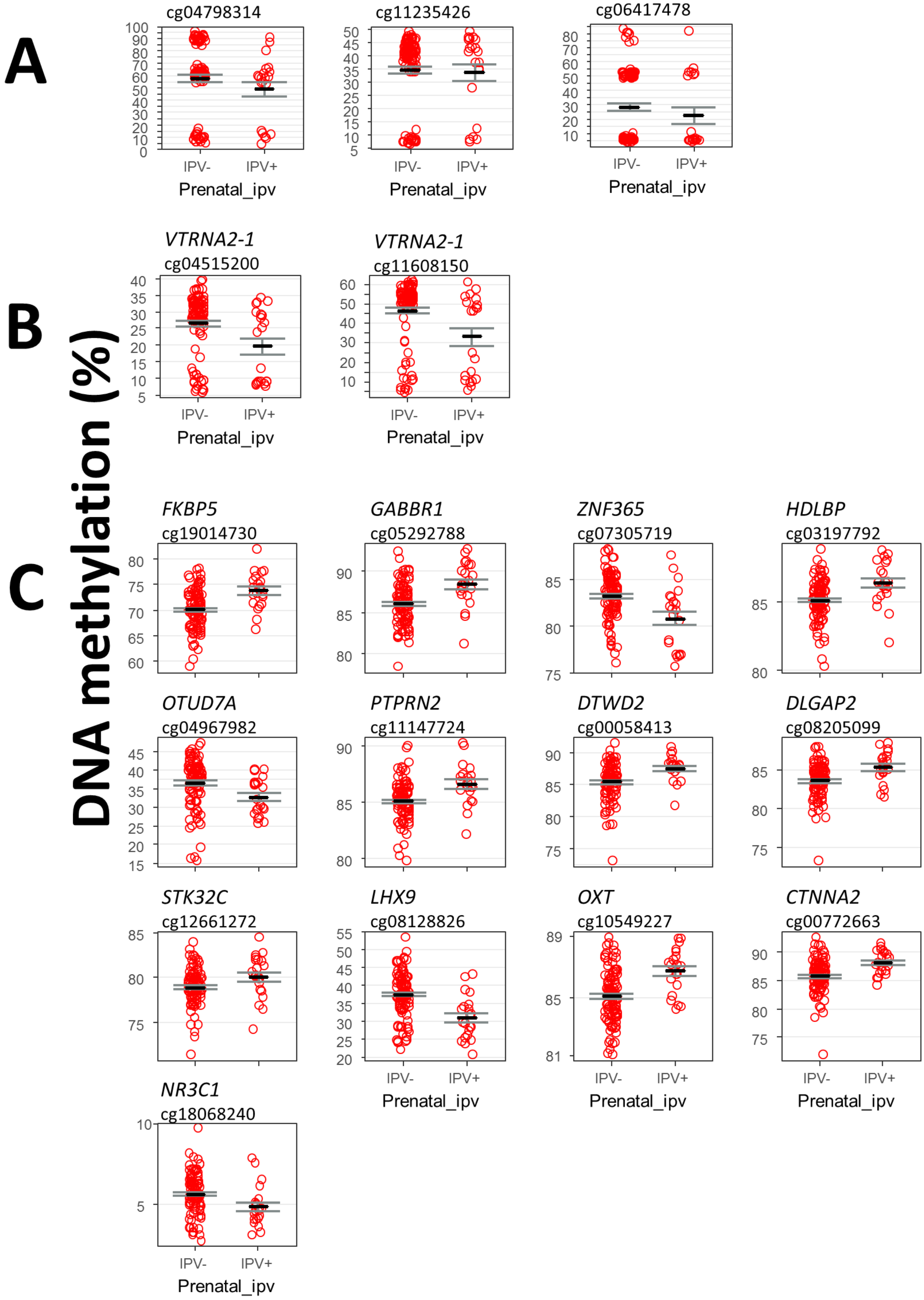

Fig S4. (Legend on next page)

Fig S4. *Local loss-of-methylation site polymorphism did not explain differences between IPV groups.* Single nucleotide polymorphism (SNP), where C have been substituted with T, A or G resulting in loss of methylation sites, may bias genome-wide methylation analysis. To minimize this possible confound, we applied two strategies. Firstly, we filtered out a list of CpGs known to harbor common SNPs (see methods for details). Secondly, we applied the robust procedure available in the limma package to our statistical models. The robust procedure is commonly used to minimize the influence of outliers in statistical models. Dot-plot panel **(A)** shows three typical examples of how genetic polymorphism in the São Gonçalo children results in methylation loss. Notice that loss of a methylation site typically leads to an averaged drop of 20-30% methylation between genotypes (homozygote C/C > 70%, heterozygote C/D ≈ 70-30%, and homozygote D/D < 30%, where D = all but C), and that genotypes typically cluster together. Also notice, that an occasional SNPs in a sample would appear as an outlier, in which the robust procedure would automatically decrease its influence on the result. Among our top candidate CpGs, only the two CpGs in the *VTRNA2-1* **(B)** showed signs of loss-of-methylation site polymorphism. Dot-plot panel **(C)** shows the 13 top candidates CpGs reported in Table 2 that previously been associated with stress or psychiatric disorders, of which none showed signs of loss-of-methylation site polymorphism. Red dots represent individual child methylation levels, while the black horizontal bar represent the group mean, and grey error bars the associated SEM (IPV– =99, IPV+ = 21).

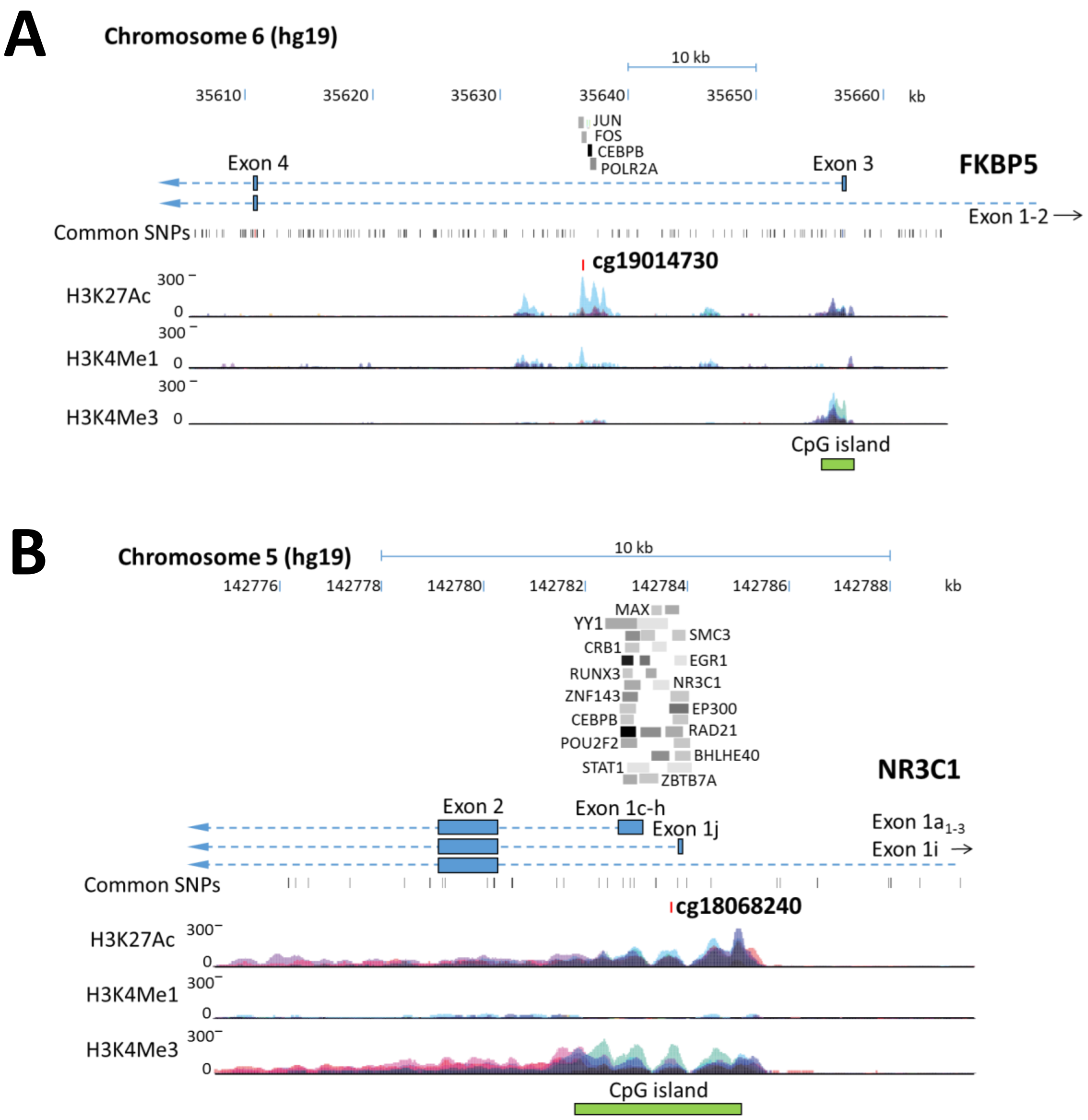

Fig S5. *Genomic context of two differentially methylated CpGs in FKBP5 and NR3C1*. Blue boxes and dotted lines in shows **(A)** *FKBP5* (cg19014730) and **(B)** *NR3C1* (cg18068240) genes. Grey/black bands represent evidence for transcription factor binding (black=stronger, grey=weaker binding) in at least one cell line, and colored peaks histone modifications from several cell lines (one color per cell line) using chromatin immunoprecipitation (ChIP). Additional information about, for instance, what cell line evidence that are available for each band/peak, graphs can be further explored at their source at the UCSC genome browser (hg19) using the default settings under the Integrated Regulation from ENCODE Tracks. Due to the bare amount of evidence for transcription factors binding in region around the CpG in *NR3C1* (cg18068240), only transcription factors with multiple evidence are reported in (B).

FKBP5:
http://genome-euro.ucsc.edu/cgi-bin/hgTracks?db=hg19&lastVirtModeType=default&lastVirtModeExtraState=&virtModeType=default&virtMode=0&nonVirtPosition=&position=chr6%3A35600690%2D35669904&hgsid=228754050_i33hAxjtfNtPBYatxnc7sMmpENgy

NR3C1:
http://genome-euro.ucsc.edu/cgi-bin/hgTracks?db=hg19&lastVirtModeType=default&lastVirtModeExtraState=&virtModeType=default&virtMode=0&nonVirtPosition=&position=chr5%3A142776579%2D142789233&hgsid=228754050_i33hAxjtfNtPBYatxnc7sMmpENgy

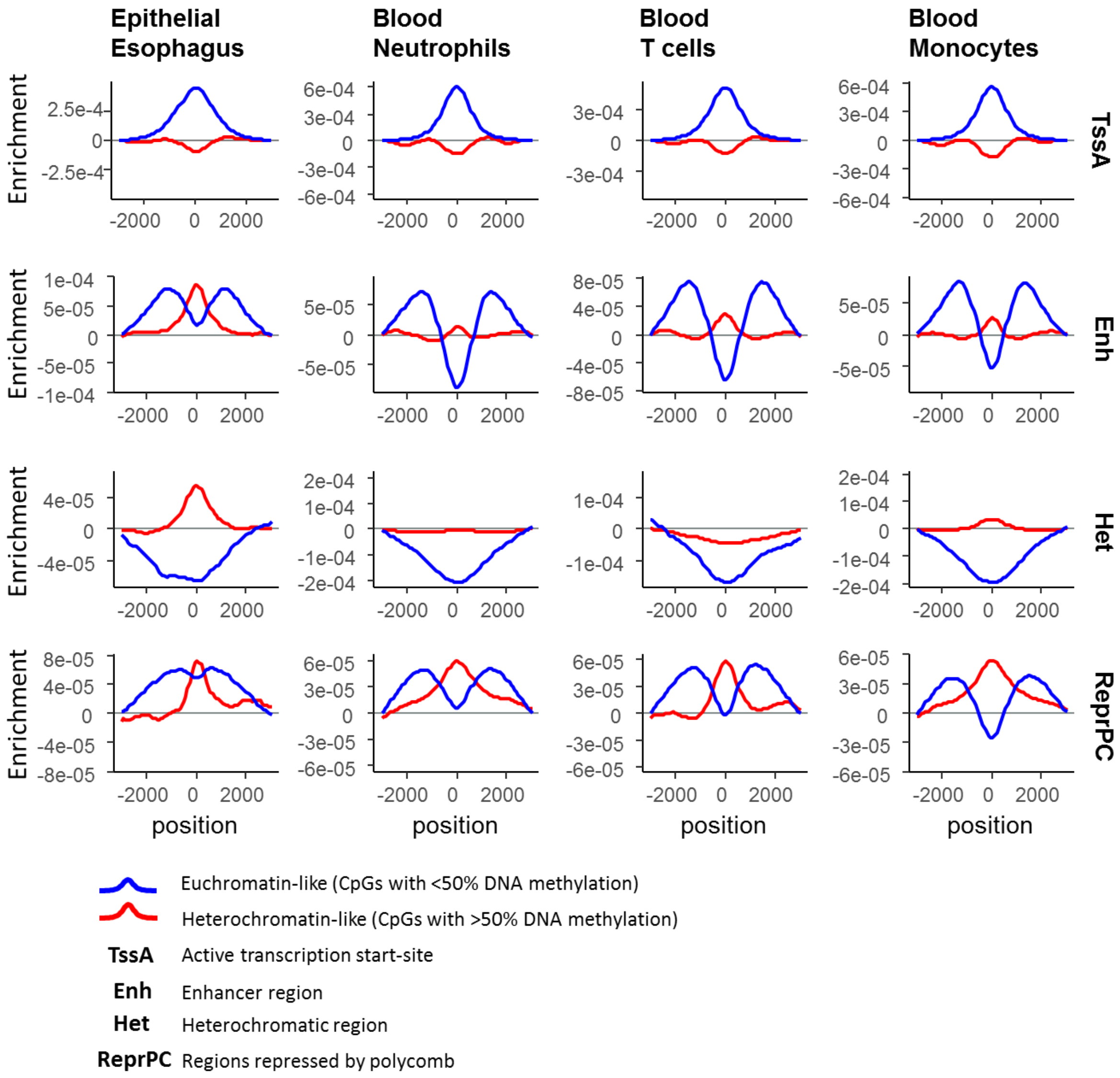

Fig S6. Chromatin state analysis using ChromHMM. Characteristics of the chromatin state in richly and poorly methylated regions. Graphs shows enrichment analyses of the overlap between chromatin states as defined by ChromHMM v1.10, and CpGs in the Euchromatin- (<50% methylation) and Heterochromatin-like (>50% methylation) regions, as identified in Fig 2C/D. We used the ChromHMM Core 15-state model that uses machine learning (a multivariate Hidden Markov Model) to categorize genomic regions based on 5 chromatin marks (H3K4me3, H3K4me1, H3K36me3, H3K27me3, H3K9me3). While ChromHMM provides chromatin states in a variety of cell-types, we only present the results from the most abundant cell-types in our saliva samples as given in Fig S2. ChromHMM Core 15 is part of the Roadmap epigenomics project and data can be found here: https://egg2.wustl.edu/roadmap/web_portal/chr_state_learning.html.

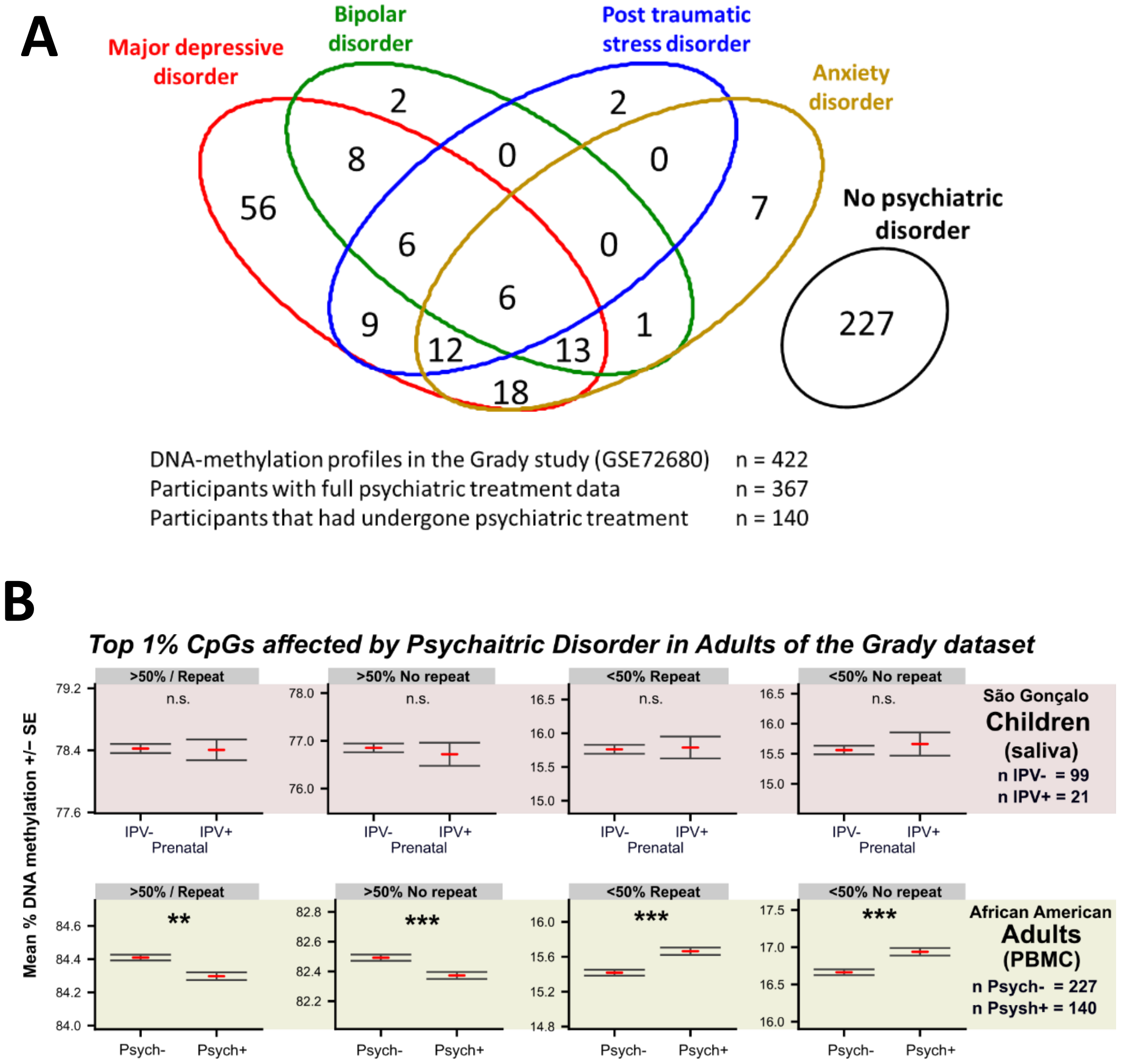

Fig S7. *A Methylome switch associated with psychiatric treatment in the Grady study.* To make an initial exploration of how methylome switching appears in diagnosed psychiatric disorders, we downloaded the freely available Grady dataset (GEO accession number: GSE72680). The Grady dataset contains Illumina HumanMethylation450 (same platform used to generate the São Gonçalo dataset) from PBMC blood cells. It also contains information whether the subjects have a history of psychiatric treatment or not. *(***A)** Shows a Wenn-diagram of the comorbidity structure of different psychiatric treatments in the Grady dataset. While treatment for Major depressive disorder was the most frequent treatment, comorbidity between psychiatric disorders was high, where 52% of the participants being treated for one disorder also had at least one secondary treatment. **(B)** Shows a methylome switch analysis of the 1% top differentially methylated CpGs between subjects with (Psych+) or without (Psych-) a history of psychiatric treatment. The graphs with yellow background show the methylome switch in the actual Grady subjects, while graphs with red background shows the same switch on the same CpGs, but in São Gonçalo children exposed (IPV+) or not exposed (IPV-) to prenatal IPV. The methylome switch analysis can be replicated from the original Grady dataset using the R script provided in Text S1 and data provided in Data S1-S4. *** p<0.001, ** p<0.01 using a General Linear Model with Sex and Age as covariates.

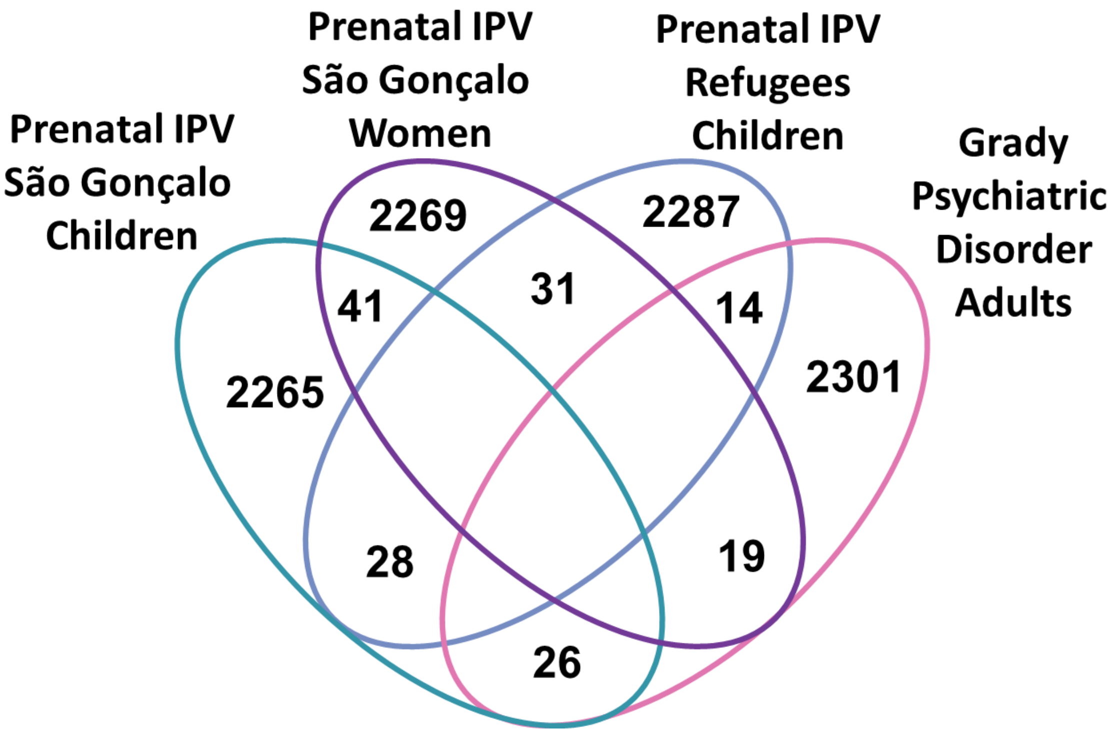

Fig S8. *Overlap between the 1% top differentially methylated CpGs across studies/populations.* Note that none of the 1% top CpGs overlapped more than two independent studies/populations. The analytical pipeline for generating the top 1% differentially methylated CpGs is available in the R script of Text S1 and can be tested using the data provided in Data S1-S4. The analysis only involved CpGs that showed enough detection signals in all datasets (detection signal p-value <0.01; see methods for details).

Table S1. Results using an extended statistical model that included additional co-variates.

CpG ID	*Chromosome*	*Position (bp)*	*Strand*	*Closest Gene*	*Log2 fold change*	*Main model* [A]			*Extended model* [B]	
						Rank	*p-value*	*FDR*	*p-value*	*FDR*
cg19014730	6	35635985	+	FKBP5	0.31	1	2.60E-07	0.05	9.43E-06	0.04
cg04967982	15	31781955	-	OTUD7A	-0.35	2	4.39E-07	0.05	1.07E-04	0.08
cg11608150	5	135415948	-	VTRNA2-1	-0.82	3	5.07E-07	0.05	2.73E-08	0.002
cg12661272	10	134054521	-	STK32C	0.16	4	1.12E-06	0.07	1.54E-05	0.05
cg02638458	7	155301811	+	CNPY1	-0.23	5	1.27E-06	0.07	2.69E-06	0.02
cg05292788	6	29586060	+	GABBR1	0.35	6	1.52E-06	0.07	3.76E-06	0.02
cg24188111	5	54603732	+	DHX29	0.2	7	1.91E-06	0.07	2.46E-06	0.02
cg24137863	11	80111021	-		0.26	8	2.40E-06	0.07	4.29E-04	0.14
cg11147724	7	157322117	-	PTPRN2	0.19	9	2.46E-06	0.07	1.63E-05	0.05
cg08857436	7	90813370	-	CDK14	0.35	10	3.20E-06	0.08	5.10E-05	0.07
cg08128826	1	197898217	+	LHX9	-0.45	11	3.61E-06	0.08	1.12E-05	0.04
cg27478707	6	32909781	+	HLA-DMB	0.28	12	3.87E-06	0.08	5.48E-04	0.15
cg17403899	19	42893709	-	CNFN	-0.22	13	4.45E-06	0.08	2.19E-05	0.05
cg21721210	19	39574878	-	ACP7	-0.29	14	4.79E-06	0.08	1.88E-05	0.05
cg04914231	16	30006637	+	INO80E	-0.37	15	5.16E-06	0.08	8.49E-05	0.08
cg07305719	10	64133406	-	ZNF365	-0.27	16	5.31E-06	0.09	6.14E-04	0.16
cg04515200	5	135415762	+	VTRNA2-1	-0.7	17	5.57E-06	0.09	2.47E-09	0.0003
cg00058413	5	118271271	+	DTWD2	0.27	18	5.87E-06	0.09	1.64E-03	n.s.
cg10549227	20	3048294	-	OXT	0.24	19	6.29E-06	0.09	3.53E-05	0.06
cg20895092	11	64933339	-	SPDYC	-0.21	20	6.43E-06	0.09	7.18E-04	0.17
cg19365706	7	100414918	+	EPHB4	0.18	21	6.51E-06	0.09	2.48E-03	0.23
cg21552292	5	14316516	+	TRIO	0.2	22	7.60E-06	0.09	3.47E-04	0.13
cg10188592	2	198364785	+	HSPE1	-0.42	23	7.62E-06	0.09	1.49E-06	0.02
cg22043788	17	80879483	-	TBCD	-0.3	24	7.81E-06	0.09	1.03E-04	0.08
cg10616337	20	5986410	-	CRLS1	-0.25	25	7.84E-06	0.09	6.57E-06	0.04
cg08108641	9	135042030	-	NTNG2	-0.22	26	9.60E-06	0.1	4.13E-04	0.14
cg03197792	2	242190985	-	HDLBP	0.18	27	1.05E-05	0.1	4.04E-05	0.07
cg08205099	8	1200896	+	DLGAP2	0.2	28	1.07E-05	0.1	7.71E-05	0.08
cg05291178	3	10149466	-	FANCD2OS	0.21	29	1.08E-05	0.1	2.48E-04	0.12
cg13494826	9	132472473	+	PRRX2	0.2	30	1.13E-05	0.1	1.54E-03	n.s.
cg00772663	2	79677079	-	CTNNA2	0.32	31	1.13E-05	0.1	2.45E-04	0.12
cg18068240	5	142783843	-	NR3C1	-0.32	39	1.76E-05	0.12	7.23E-06	0.04

[A] The main model used in the analysis for finding differentially methylated CpGs (used in Table 2):
Meth = IPV + Age + Sex + CDV + trauma

[B] Data analyzed using an extended model with additional covariates:
Meth = IPV + age + sex + CDV + trauma + income + addiction + chip + CD4 + CD8 + Nue + mono + Bcells + NK + saliva + buccal + epi

Meth	Methylation level (Y)
IPV	Prenatal IPV+ vs IPV- (X)
age	Reported age (Table 1; Fig S3)
sex	Sex (Table 1)
CDV	Prenatal community domestic violence score (Table 1)
trauma	Prenatal trauma scores (Table 1)
income	Family income (Table 1)
addiction	Maternal smoking or alcohol use (Table 1)
chip	Chip ID number (each bead chip contained 12 arrays)
CD4-NK	Factor scores from the Houseman blood cell reference analysis (Fig S2)
saliva	Factor scores from custom saliva reference (Fig S2)
buccal	Factor scores from custom buccal reference (Fig S2)
epi	Factor scores from the Epistructure genetic structure analysis (Fig S2)

Table S2. Results and primer sequences for the validation of Infinium HumanMethylation450K BeadChip using targeted bisulfite sequencing.

	Yield bisulfite sequencing					*Comparsion with array target CpGs*	
Sample	*Mapped reads*	*Mapping Efficiency*	*n CpGs covered*	*Avg. CpG Coverage*	*Bisulfite Conversion*	*Pearson correlation*	*P-value*
S01	69761	66%	108	5591X	>99%	0.96	<0.0001
S02	58014	71%	108	4853X	>99%	0.94	<0.0001
S03	81533	74%	108	6107X	>99%	0.94	<0.001
S04	73717	73%	108	5823X	>99%	0.94	<0.0001
S05	64396	72%	108	5061X	>99%	0.92	<0.001
S06	66885	72%	108	5448X	>99%	0.93	<0.001
S07	86226	69%	108	6331X	>99%	0.96	<0.0001
S08	99207	77%	108	7488X	>99%	0.91	<0.001
S09	67465	74%	108	5238X	>99%	0.94	<0.0001
S10	54759	74%	108	3963X	>99%	0.95	<0.0001
S11	54296	72%	108	4426X	>99%	0.93	<0.001
S12	52821	72%	108	4128X	>99%	0.96	<0.0001
S13	71285	70%	108	5661X	>99%	0.93	<0.0001
S14	71832	73%	108	5632X	>99%	0.92	<0.001
S15	70161	71%	108	5708X	>99%	0.95	<0.0001
S16	67824	75%	99	4176X	>99%	0.90	<0.001
S17	62476	77%	108	4645X	>99%	0.96	<0.0001
S18	72547	67%	108	6046X	>99%	0.94	<0.0001
S19	63013	67%	108	5001X	>99%	0.94	<0.0001
S20	62125	71%	108	5185X	>99%	0.96	<0.00001
S21	66159	71%	108	5769X	>99%	0.93	<0.001
S22	65391	69%	108	5063X	>99%	0.93	<0.0001
S23	90698	72%	108	6470X	>99%	0.93	<0.001
S24	59314	70%	108	4593X	>99%	0.96	<0.0001
S25	65629	70%	108	5160X	>99%	0.94	<0.0001
S26	46640	66%	108	3803X	>99%	0.93	<0.001
S27	67222	69%	108	5126X	>99%	0.95	<0.0001
S28	67466	67%	108	5562X	>99%	0.94	<0.0001
S29	62425	74%	108	4896X	>99%	0.93	<0.001
S30	88432	74%	108	6652X	>99%	0.95	<0.0001
S31	68868	69%	108	5798X	>99%	0.91	<0.001
S32	56563	72%	108	4233X	>99%	0.91	<0.001
S33	44262	73%	92	3598X	>99%	0.92	<0.001

Note: Assays were ordered at Zymo Research using their Targeted Sequencing for DNA Methylation Analysis

Primer sequences:

Target: cg18068240/chr5_142783844 Read coverage: 4664±180 SEM Region: chr5:142783644-142783943 Fwd: TYGTTTTTTTTTTGAAGTTTTTTTAGAGGG Rev: AAAACTCRCTCTACCCCTTAAC
Target: cg17403899/chr19_42893710 Read coverage: 1358±158 SEM Region: chr19:42893510-42893809 Fwd: GGATAGATTTTGTTATTGTTGTTATAGTT Rev: CATTTCTAAAACCTCTAAAATAAAAACAAA
Target: cg08128826/chr1_197898217 Read coverage: 2961±217 SEM Region: chr1:197898067-197898366 Fwd: TAATTGGGATTGGTTTGTAGGTTTGGTTTTA Rev: ATCACTACAATAATAATAAAATTAATCAAATCTATT
Target: cg09963640/chr18_31157661 Read coverage: 14376±550 SEM Region: chr18:31157511-31157810 Fwd: TTTATGTGTATTATGTTTTATTTTATAGAATGTTGA Rev: TACAACTTAACACAATATTAAACCACCTACAA
Target: cg10516336/chr3_183735216 Read coverage: 1825±115 SEM Region: chr3:183735166-183735465 Fwd: GAGTTATTYGTTGTATAGTTTTTTAGGTGG Rev: CRCCTCCTAAACCTATAAACCC
Target: cg21212505/chr7_117228958 Read coverage: 4084±173 SEM Region: chr7:117228808-117229107 Fwd: AGGTTGTTAGATGTTAATTTGTTAAAAGTTA Rev: AATCAACTTCCATTTATTTAAAAACCTATAAATTAT
Target: cg06260077/chr11_27721350 Read coverage: 789±32 SEM Region: chr11:27721150-27721449 Fwd: GTTTATTTTAGTTTTAATTTTTGGGGAGTAGGTAG Rev: CCCTCCCCCAACCCAAAAATAA
Target: cg01311051/chr16_30064897 Read coverage: 311±17 SEM Region: chr16:30064747-30065046 Fwd: TTTTYGGATTGATTGGTTTTGTTTTTTTTTATG Rev: TTAATCAACTCCTTATCTTACCAAAAATACC
Target: cg19014730/chr6_35635985 Read coverage: 2141±88 SEM Region: chr6:35635835-35636134 Fwd: TTTTATGGTAATAGTAGATTAGTTATTGTATAAAA Rev: TCCCRAAATACTATTTCCAACAT
Target: cg12661272/chr10_134054522 Read coverage: 34840±1245 SEM Region: chr10:134054372-134054671 Fwd: TGTTTTYGTTGATTTTTGTAGGGGTAGA Rev: CAAAAAACTCTCRAACCCCCAAAA
