# Supplementary material for: Does Prenatal Stress Shape Postnatal Resilience? – An Epigenome-Wide Study on Violence and Mental Health in Humans
Source: Front Genet. 2019 Apr 16;10:269. doi: 10.3389/fgene.2019.00269 (PMC6477038; doi:10.3389/fgene.2019.00269)
Supplement: Supplementary file 2 — Datasheet 2.zip contains the script and data for reproducing some of the analysis of this paper, this includes: Supplementary Text S1 which contains the R script that will reproduce our analysis using Supplementary Data S1–S4; Supplementary Text S2 explains the column names in Supplementary Data S1; Supplementary Data S1 is the result table with beta-values for the top 1 % most differentially methylated CpGs between IPV+ and IPV- São Gonçalo children and women; Supplementary Data S2 contains the factors and co-variates for São Gonçalo children and women; Supplementary Data S3 contains the factors and co-variates for the Grady data downloaded from Gene Expression Omnibus; Supplementary Data S4 contains a list of excluded CpGs/probes as described in the Materials and Methods. For possible updates to the R script in Supplementary Text S1 check https://github.com/Danis102 or contact the corresponding author. [file Data_Sheet_2.zip › Text_S2-Column_descriptions.pdf]

Explanations to column names in Data S1

Column name	Description
chr	Chromsome
pos	Genomic coordinate
strand.x	DNA strand
Name	Illumina CpG ID
UCSC_RefGene_Name	Gene symbol as in UCSC browser
logFC_MvalC	Log2 fold changes of M-values in children (prenatal IPV+ vs prenatal IPV-)
AveExpr_MvalC	Average expression after limma normalization in children (prenatal IPV+ vs prenatal IPV-)
P.Value_MvalC	Original p-value generated in limma in children (prenatal IPV+ vs prenatal IPV-)
adj.P.Val_MvalC	Benjamini–Hochberg corrected p-values in children (prenatal IPV+ vs prenatal IPV-)
logFC_MvalW	Log2 fold changes of M-values in women (prenatal IPV+ vs prenatal IPV-)
AveExpr_MvalW	Average expression after limma normalization in women (prenatal IPV+ vs prenatal IPV-)
P.Value_MvalW	Original p-value generated in limma in women (prenatal IPV+ vs prenatal IPV-)
adj.P.Val_MvalW	Benjamini–Hochberg corrected p-values in women (prenatal IPV+ vs prenatal IPV-)
logFC_MvalM	Log2 fold changes of M-values in mothers (prenatal IPV+ vs prenatal IPV-)
AveExpr_MvalM	Average expression after limma normalization in mothers(prenatal IPV+ vs prenatal IPV-)
P.Value_MvalM	Original p-value generated in limma in mothers (prenatal IPV+ vs prenatal IPV-)
adj.P.Val_MvalM	Benjamini–Hochberg corrected p-values in mothers (pregnancy IPV+ vs pregnancy IPV-)
logFC_BvalC	Same as logFC_MvalC but using beta-values as input to limma
AveExpr_BvalC	Same AveExpr_MvalC but using beta-values as input to limma
P.Value_BvalC	Same P.Value_MvalC but using beta-values as input to limma
adj.P.Val_BvalC	Same adj.P.Val_MvalC but using beta-values as input to limma
Column 23-142 ("_child")	Normalized beta values (percent DNA methylation) of individual children
Column 143-264 ("_woman")	Normalized beta values (percent DNA methylation) of individual woman
Islands_Name	Illumina annotation: CpG island overlapping the CpG (genomic coordinates)
Relation_to_Island	Illumina annotation: CpG location in relation to CpG island
UCSC_RefGene_Accession	Illumina annotation: Reference numbers
UCSC_RefGene_Group	Illumina annotation: CpG location in relation to gene
DMR	Illumina annotation: Previously described differentially methylated region
Enhancer	Illumina annotation: Overlapping with enhancer region
Regulatory_Feature_Name	Illumina annotation: Other regulatory feature.
Regulatory_Feature_Group	Illumina annotation: Other regulatory feature classification
seqnames.ext.	Genomic coordinates +/-50 bp from CpG.
start.ext.	Genomic coordinates +/-50 bp from CpG.
end.ext.	Genomic coordinates +/-50 bp from CpG.
Repeat	Repeatmasker annoation: Overlap repetitative region +/-50 bp
repClass	Repeatmasker annoation: Repeat class +/-50 bp
repFamily	Repeatmasker annoation: Repeat family +/-50 bp
repName	Repeatmasker annoation: Repeat name +/-50 bp
seqnames	Other overlapping feature within +/- 50bp: Feature genomic coordinates
start	Other overlapping feature within +/- 50bp: Feature genomic coordinates
end	Other overlapping feature within +/- 50bp: Feature genomic coordinates
width	Other overlapping feature within +/- 50bp: Feature genomic width
CpGi	Other overlapping feature within +/- 50bp: Overlapping CpG island as defined by UCSC
exon.width	Other overlapping feature within +/- 50bp: Overlapping exon width as defined by UCSC
exon_rank	Other overlapping feature within +/- 50bp: Overlapping exon rank in order as defined by UCSC
exon_tx.tot.num	Other overlapping feature within +/- 50bp: Number of exons in gene as defined by UCSC
exon	Other overlapping feature within +/- 50bp: Overlapping exon as defined by UCSC
Braz_c_1perc	Among the 1% top ranked in São Gonçalo children (prenatal IPV+ vs prenatal IPV-)
Braz_w_1perc	Among the 1% top ranked in São Gonçalo women (prenatal IPV+ vs prenatal IPV-)
Grady_1perc	Among the 1% top ranked in Grady study (psychiatric treatment+ vs psychiatric treatment-)
